# AI-augmented decision-making in face matching: comparing concurrent and non-concurrent advice presentation

**DOI:** 10.1186/s41235-026-00707-z

**Published:** 2026-02-05

**Authors:** Eesha Kokje, Eva Lermer, Anne-Kathrin Kleine, Susanne Gaube

**Affiliations:** 1 Center for Leadership and People Management, LMU Munich, Giselastr. 10, 80802 Munich, Germany; 2 Department of Business Psychology, Technical University of Applied Sciences Augsburg, Augsburg, Germany; 3 Global Business School for Health, UCL, London, UK

**Keywords:** Human–AI interaction, Face matching, Artificial intelligence, Decision-making, AI advice, Overreliance

## Abstract

A primary aim of human–AI teaming is to achieve better collaborative performance than either can achieve alone. Despite considerable efforts in this direction, issues such as overreliance of users on decision aids continue to be a challenge which prevent this. In this study, we evaluated the potential of non-concurrent advice presentation as a strategy to reduce overreliance in a face-matching task. We conducted three pre-registered experiments examining (a) on-demand binary advice, (b) on-demand similarity ratings, and (c) conditional advice (i.e. advice presented only if participants’ initial unaided decision is different from the AI prediction), compared to concurrent advice. Across all experiments, we did not find significant differences in the overall performance of participants in the concurrent vs. experimental conditions. But, we found that participants followed AI advice more when they demanded it. Conversely, when they demanded similarity ratings, they followed advice less. Thus on-demand similarity ratings reduced overreliance on AI compared to concurrent similarity ratings presentation. However, overall, similarity ratings were not more helpful compared to basic advice. We also found that participants were less likely to follow AI advice when presented after their initial unaided decision contradicted the AI prediction and were more confident in rejecting incorrect advice, but not as confident when accepting correct advice. Overall, non-concurrent paradigms have potential to reduce overreliance, but at the cost of underreliance on correct advice.

**Supplementary Information:**

The online version contains supplementary material available at 10.1186/s41235-026-00707-z.

## Significance statement

As AI-based tools become more common in everyday decision-making, it is crucial to understand how the human user interacts with and utilizes the input received from the AI. One issue that is a continuing challenge in the effective integration of AI tools is overreliance of users on AI. As highly accurate AI tools, too, are susceptible to errors, overreliance on these tools can be detrimental to human-AI collaboration. Therefore, it is important to identify appropriate methods and designs that can help users accurately calibrate their level of trust and reliance on AI tools. Using a one-to-one face matching paradigm, our study tested whether changing when people see AI advice, such as, only showing advice when the user demands it or when there is an incongruency between the AI’s judgement and the decision made by the human, could reduce the problem of overreliance. Our findings suggest that delaying the input from the AI and showing it selectively can reduce overreliance, but it also reduces overall reliance on the AI, meaning that users also disregard correct advice more frequently. Therefore, in terms of concurrent vs. non-concurrent advice presentation, there appears to be no one-size-fits-all solution; rather, it is essential to consider the context and use case in order to determine the most appropriate design for human-AI interaction.

**Supplementary Information:**

The online version contains supplementary material available at 10.1186/s41235-026-00707-z.

## Introduction

The rapid developments in the field of Artificial Intelligence (AI) have resulted in its increased deployment in AI-based Decision Support Systems (AI-DSS) in various fields (Higgins et al., 2023; Rajpurkar et al., 2022; Yousaf et al., 2023). Although AI technology is improving rapidly, with accuracy rates of many algorithms outperforming humans (Agarwal et al., 2024; Choi & Schwarcz, 2023; Jansen et al., 2024; Yoon et al., 2023), their effective integration as decision aids to humans has been less than ideal (Steyvers & Kumar, 2024). While AI-DSSs have the potential to enhance decision accuracy and efficiency, they also bring challenges arising from humans and AI having to collaborate, including risks of overreliance (i.e. when users accept incorrect AI advice), distrust in insights generated by AI, and difficulty interpreting the insights (Cohen et al., 2023; Klingbeil et al., 2024). As long as these AI systems do not make autonomous decisions without a human in the loop, their effectiveness ultimately depends on their users, and how well they are able to engage with the information derived from the system, and thereby on how the information is conveyed (Yang et al., 2020). Therefore, understanding how different aspects of human–AI interaction impact decision-making is crucial for designing AI systems that effectively support humans.

A major challenge in human–AI collaboration is *automation bias,* which is the tendency of users to favour automated decision support systems while ignoring contradictory information (Goddard et al., 2011; Skitka et al., 1999). One type of error introduced by automation bias is overreliance on the AI-DSS, wherein the user accepts incorrect AI advice, as they may find it difficult to discern whether advice is correct or incorrect (Passi & Vorvoreanu, 2022). A potential solution in the form of explainability, wherein additional information about the AI’s prediction is provided, has been proposed to overcome this issue (Vasconcelos et al., 2023) but has shown very mixed results (Zhang et al., 2024). While some studies show some improvement in performance with explainable advice (Gaube et al., 2024; Senoner et al., 2024), other studies show either no effect (Bertrand et al., 2023; Cecil et al., 2024) or even a negative impact, wherein overreliance increases when explanations are provided (Jacobs et al., 2021; Schemmer et al., 2022), as the presence of an explanation itself increases trust (Bertrand et al., 2022). Therefore, we need to examine potential solutions beyond explainability.

The presentation of advice affects how individuals perceive, trust, and act upon AI-generated suggestions (Buçinca et al., 2021; Zhang et al., 2020). One important aspect is the sequence or timing of advice presentation, as users may weigh AI advice differently based on when it is received in the decision process (Buçinca et al., 2021; Fogliato et al., 2022). If advice is presented concurrently, users may be more open to the advice, but their engagement with the task may be more superficial (Gajos & Mamykina, 2022) and their response may be biased by the advice due to the *anchoring effect* (Furnham & Boo, 2011). The anchoring effect refers to a cognitive bias which causes our decisions or judgements to be influenced by an initial reference point (in this case, the AI advice), which may result in overreliance (Buçinca et al., 2021). Studies consistently show that incorrect AI advice proves more detrimental to decision performance than not receiving advice (Gaube et al., 2024; Klingbeil et al., 2024; Kokje et al., 2024). If advice is presented with a delay, it might give the user a chance to first engage with the task independently without being influenced by the AI’s judgement and decide for themselves if AI input would be helpful (Buçinca et al., 2021; Kumar et al., 2021; Liang et al., 2022; Papenkordt, 2024; Park et al., 2019). Consequently, overreliance on incorrect advice may be reduced if a non-concurrent presentation approach was used, which could be (a) leaving users to decide if they require assistance (i.e. advice on-demand) or (b) only presenting advice in case of a disagreement between the human’s decision and the AI’s prediction. The current study aims to investigate whether non-concurrent advice presentation can reduce overreliance, in a one-to-one face-matching paradigm.

### AI-DSS in face identification

The use of photo IDs for identity verification is common in a variety of applied settings, ranging from settings such as border controls to ID checks at supermarkets for buying age-restricted products. This typically involves verifying whether the face on the photo ID picture matches the face of the individual carrying it, in what is known as one-to-one face matching. The use of AI-based technology for this purpose has been steadily increasing (Gupta et al., 2023), particularly as these systems become increasingly accurate, surpassing human performance and some achieving near-perfect accuracy under ideal conditions (Grother et al., 2024; Phillips et al., 2018). However, despite their impressive performance, these systems are still susceptible to errors, and therefore, require human oversight (Hancock et al., 2020). Previous studies comparing human and algorithm performance on unfamiliar face-matching tasks reported that fusing similarity judgements of humans and algorithms leads to better accuracy than humans alone or algorithms alone (O’Toole et al., 2007; Phillips et al., 2018). Phillips et al. (2018), particularly, showed that fusing the judgements of one facial examiner and an algorithm resulted in perfect accuracy. This gives impetus for combining human and AI judgements through AI-DSSs and for examining appropriate design and presentation methods that would lead to better accuracy.

Several studies have emerged in recent years that have examined the influence of specific human, AI, and design aspects on AI-augmented human performance in face-matching tasks. Howard et al. (2020) examined the impact of the *source of advice* and found that participants trusted the AI’s judgement more than another human’s judgement. However, the difference in trust was not reflected in their performance, which was similar for AI-aided and human-aided decisions. Contrary to that, Carragher et al.(2024) reported that participants who showed generally higher trust in automation, as well as participants who trusted AI’s judgement over another human’s judgement specifically in a face-matching task, showed greater gains in AI-aided performance. Additionally, Howard et al., (2020) recorded participants’ responses on a 7-point certainty scale and reported that presenting AI’s judgement shifted participants’ response certainty in the direction of the AI judgement. Fysh and Bindemann (2018) similarly reported that participants attend to and incorporate AI judgements into their decisions, even when they are instructed to ignore them. Moreover, when participants were provided feedback on the accuracy of their decisions (i.e. participants were informed after each trial whether their response was correct or incorrect), positive experiences with the AI (i.e. repeated instances where the AI advice was correct) resulted in an increased reliance on AI advice.

Further, a study by Carragher and Hancock (2023) tested the effect of the *accuracy rate of the algorithm* and found that participants tend to adjust their reliance on the system according to the accuracy of the system, even when participants are not informed of the accuracy rate. Another study, by Kokje et al. (2024), manipulated the *perceived accuracy of the AI* (i.e. the real accuracy rate remained uniform throughout, but participants were told different rates). Participants adjusted their reliance on the AI even when real accuracy remained unchanged, as they tended to follow AI predictions more when a high-accuracy rate was implied, and they followed AI predictions less when a low accuracy rate was implied.

A common finding across the studies was that incorrect advice significantly worsens performance not only compared to when correct advice is presented, but also compared to when no advice is presented. This is due to the previously discussed issue of overreliance on AI systems. Two studies (Carragher & Hancock, 2023; Kokje et al., 2024) provided additional information in the form of face similarity scores in addition to the binary match/mismatch judgements, while one study (Mueller et al., 2024) provided only similarity scores or only binary match/mismatch judgements, to evaluate whether this reduces overreliance. Carragher and Hancock (2023), as well as Mueller et al. (2024) reported no difference in overall performance in the two conditions, while Kokje et al. (2024) found a marginal improvement in performance with the similarity ratings. However, this was observed only when the advice was correct. Consequently, similarity ratings did not reduce overreliance on incorrect advice in any of the studies.

As discussed previously, non-concurrent advice presentation strategies may potentially reduce bias resulting from seeing advice before engaging with the task, and thereby overreliance too. Therefore, in the current study, we examined the potential of different strategies of non-concurrent advice presentation in reducing overreliance on AI advice and improving performance. The study aimed to address the following questions:

1. Do non-concurrent advice presentation strategies help to reduce overreliance on AI advice compared to concurrent advice presentation?
2. Do similarity ratings have a more positive impact when their presentation is delayed and provided selectively compared to when presented concurrently and compared to binary advice?
3. Do non-concurrent advice presentation strategies improve overall performance of the human–AI team compared to concurrent advice presentation strategies? Does the human–AI team performance exceed that of the AI alone?

We conducted three pre-registered experiments, in which participants completed a one-to-one face-matching task which involved making decisions about whether face pairs belonged to the same person or to two different people. In each experiment, we manipulated when and under which condition AI advice was presented to users in the decision-making process, to assess its impact on users’ reliance on the AI system and performance of the human–AI team. In the first two experiments, we tested on-demand binary advice (Experiment 1a) and on-demand similarity ratings (Experiment 1b); and in the final experiment, we tested the influence of providing advice only after an initial decision is made and if that decision contradicts the AI’s prediction. Essentially, across all experiments, we compared performance and reliance when binary advice (or binary advice + similarity rating) is provided by default and simultaneously with the stimuli vs. when binary advice (or similarity rating) presentation is delayed and provided optionally.

## General method

The experiments consisted of a one-to-one face-matching task, which involved two faces presented simultaneously on a screen. The faces either belonged to the same person (match) or to different people (mismatch). Participants were asked to make match or mismatch decisions for each face pair. The experiments were pre-registered on OSF (Experiment 1a, Experiment 1b, Experiment 2). The study received ethical approval from the Joint Ethics Committee of Bavarian Universities (GEHBa-202405-V-193-R).

### Stimuli

**Fig. 1:**
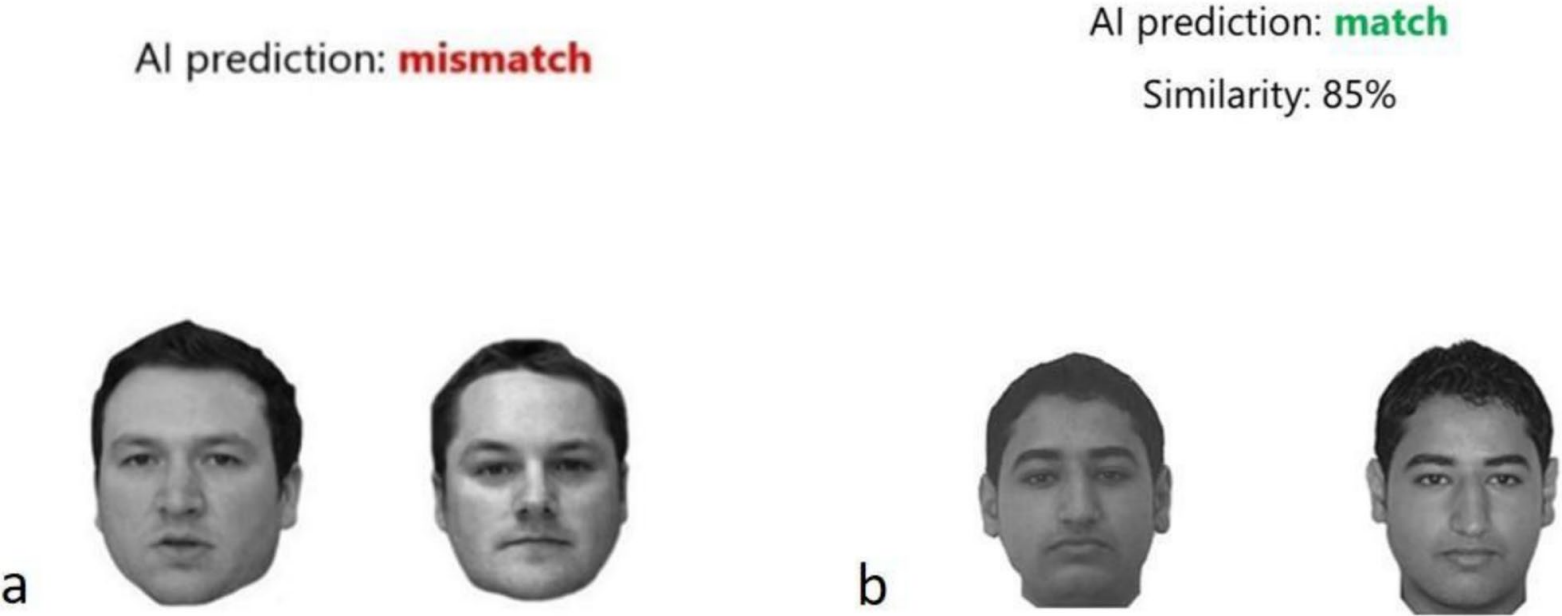
Example stimuli. The example depicts (**a**) a mismatch pair (from the GUFD) with binary advice and (**b**) a match pair (from the Arab face database) with binary advice and similarity rating

Stimuli consisted of face pairs in frontal view presented in greyscale without background information (see Fig. 1 for example stimuli). It was ensured that only the head and face were visible by removing all external background information removed, i.e. clothing, accessories, or background. The faces showed a neutral expression. We took 100 face pairs from the Glasgow University Face Database (GUFD), which contained images of Caucasian faces (Burton et al., 2010), and 100 face pairs from an Arab database containing images of Arab faces (Megreya & Burton, 2008). Both followed the same style for constructing the stimuli. All face pairs were male, as female faces for the Arab database were not available. Half of the face pairs in each database were match cases (i.e. depicted the same person, and half were mismatch cases (i.e. depicted different people). The methods for the construction of the face arrays are detailed in the original studies (Burton et al., 2010; Megreya & Burton, 2008).

An AI prediction was generated for each face pair with deepface (https://github.com/serengil/deepface), using Facenet—an openly available deep convolutional neural network AI model (Schroff et al., 2015). Further details on the face pairs and the AI predictions are presented in the supplementary materials. A binary prediction of ‘match’ or ‘mismatch’ was presented. In cases where additional information regarding the binary match/mismatch classification was to be presented, we presented similarity ratings. To obtain these, we inverted the distance value calculated by the algorithm to a similarity value (1-distance) and expressed it as a percentage, for ease of understanding. For example, if the distance value calculated by the algorithm was 0.75, this was inverted to a similarity value of 25%. The AI predictions were accurate in 92.5% of the cases presented. For the purpose of clarity, from here on, we distinguish between *AI predictions* and *AI advice*. *AI advice* refers specifically to cases where participants actually saw the binary AI prediction, whereas *AI prediction* refers to prediction generated by the AI, which the participant may or may not have seen.

The stimuli were divided and presented in three or four blocks with 40 face pairs (i.e. trials) in each block. Each block contained the same ratio of accurate and inaccurate AI advice cases in order to maintain the accuracy rate at 92.5%.

### Procedure

The experiments were programmed in Psychopy (Peirce, 2007), and the study was conducted online via Pavlovia. Participants were recruited over Prolific and were living in Germany. They received monetary compensation (~ £9/hr) for their time. Participants gave informed consent and began by completing a short demographic questionnaire over Qualtrics, followed by general instructions about the task and each of the conditions presented within that experiment. Additionally, participants were informed that the top 10% of performers would receive a bonus payment to incentivize them to perform to the best of their ability. Participants were also informed that the AI prediction could be inaccurate in some cases, but the overall accuracy rate was not provided. Instructions, along with practice trials, were provided at the beginning of each condition, followed by the block of trials. A short break was provided at the end of each block.

Participants’ task was to make a ‘match’ or ‘mismatch’ decision for each face pair and register that decision with a keypress. In each block, an attention check item was added in the form of a face pair consisting of a male and a female face (an obvious mismatch case), with the AI prediction showing a ‘match’.

### Dependent variables

*Performance* was measured as the percentage of correct decisions, based on participants’ match/mismatch responses, which were binarily coded as correct (1) or incorrect (0).

*AI agreement* was the percentage of responses where participants’ responses matched the AI prediction.

### Statistical analyses

The analysis was performed using RStudio (Version 2023.09.1 + 494). Participants were excluded from the data analysis if they failed two or more attention checks, if they did not finish the experiment, if they completed the study in an unrealistically short time (avg. RT < 1 s), or if their performance was below chance-level (50%). We conducted mixed-effects logistic regression analysis for ‘performance’ and ‘AI agreement’ measures, with face pairs and participants as random factors. Fixed factors are specified individually in each experiment.

## Experiment 1a

In this experiment, we tested the effect of presenting AI advice only when participants request it (*on-demand condition*) and compared performance to when advice is provided concurrently with the stimuli (*concurrent condition*).

### Participants

Eighty individuals completed the study and were included in the final analysis. Demographic characteristics of our sample from Experiment 1a are presented in Table 1.

**Table 1:** Participant characteristics in Experiment 1a

	(*N* = 80)
*Age*	
Mean (SD)	31.0 (10.3)
*Gender*	
Female	21 (26.3%)
Male	57 (71.3%)
Non-binary	2 (2.5%)
*Ethnicity*	
Black/African American	1 (1.3%)
Hispanic/Latin American	1 (1.3%)
Middle Eastern/Arab	4 (5.0%)
Turkish	2 (2.5%)
White/Caucasian	72 (90.0%)

### Stimuli and procedure

Participants were exposed to two conditions. In the *concurrent advice condition,* the binary AI prediction was presented concurrently with each face pair. In the *on-demand advice condition*, face pairs were initially presented without the AI prediction, but participants could choose to view the AI prediction with a keypress. The stimuli remained on the screen until the end of each trial, even when participants chose to view the AI prediction, until they registered a match or mismatch response. Once they registered their decision, with or without viewing the AI prediction, the experiment continued with the subsequent trial. Participants did not receive feedback on their decisions.

Participants were presented with 160 face pairs divided into four blocks. Two blocks were paired with the *concurrent advice condition* and two blocks with the *on-demand advice condition*. Two versions of the experiment were created to counterbalance the order of presentation of the two conditions and to ensure that each face pair was paired with both conditions. Each participant completed only one version.

### Results and discussion

**Fig. 2:**
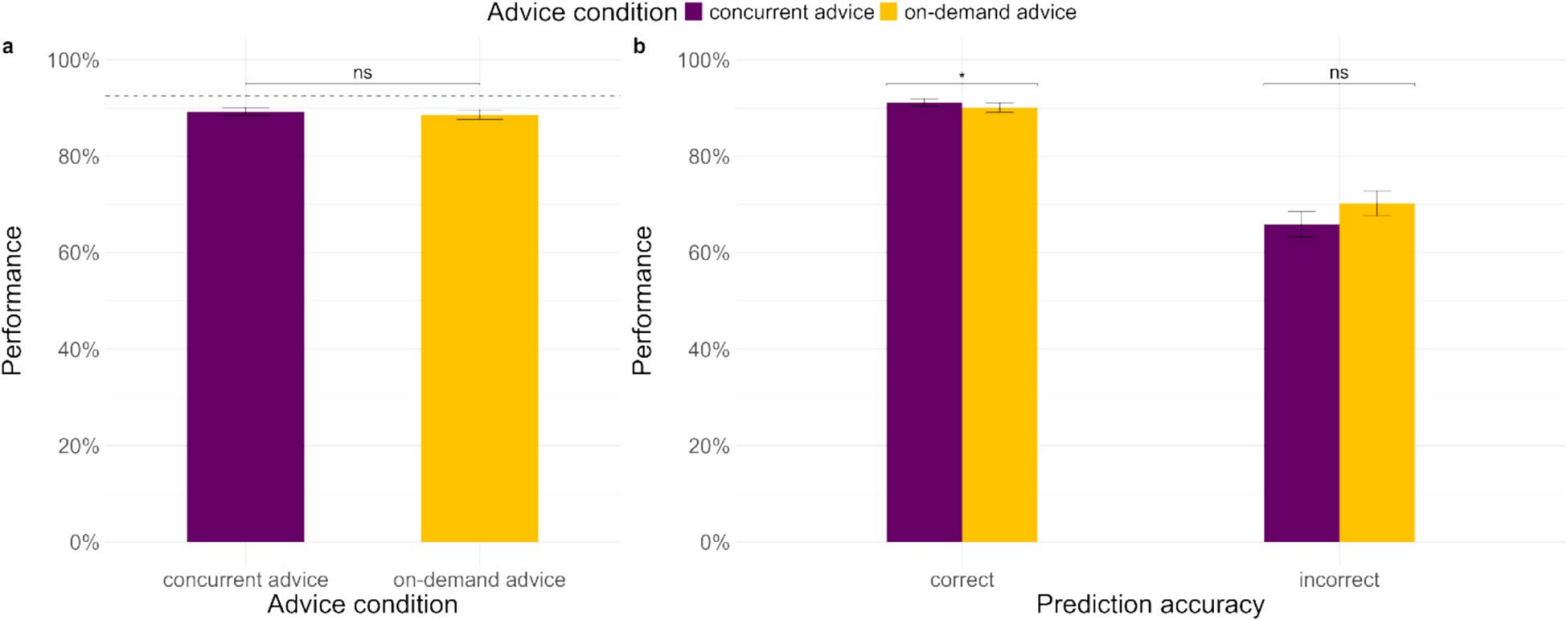
Performance on the task (**a**) overall in the two advice conditions (dotted line represents AI’s accuracy level), and (**b**) the advice conditions by the accuracy of the prediction

We first examined the overall performance of participants in the *concurrent advice condition* and the *on-demand advice condition* (see Fig. 2a). The mixed-effects logistic regression with advice condition as a fixed factor showed that overall performance was not influenced by advice condition (see Table 2). We further examined the interaction of advice condition with the accuracy of the AI prediction (see Table 2), which showed that performance was significantly better in the concurrent advice condition for correct prediction trials (see Fig. 2b). In case of incorrect prediction trials, performance was somewhat better in the on-demand advice condition, but not significantly so. It is possible that the incorrect prediction trials lacked power.

**Table 2:** Logistic mixed-effects regression models for participants’ performance in Experiment 1a

Predictors	Performance					Performance					Performance				
	Odds Ratios	SE	95% CI	Statistic	*p*	Odds Ratios	SE	95% CI	Statistic	*p*	Odds Ratios	SE	95% CI	Statistic	*p*
Intercept	18.15	2.74	13.49 – 24.41	19.17	** < 0.001**	21.58	3.22	16.11 – 28.91	20.60	** < 0.001**	22.03	3.33	16.39 – 29.61	20.48	** < 0.001**
Advice condition [on-demand]	0.93	0.06	0.82 – 1.05	-1.14	0.255	0.87	0.06	0.76 – 1.00	-1.97	**0.049**					
Prediction accuracy [incorrect]						0.11	0.04	0.05 – 0.23	-5.62	** < 0.001**	0.10	0.04	0.05 – 0.23	-5.57	** < 0.001**
Advice condition [on-demand] x Prediction accuracy [incorrect]						1.61	0.31	1.10 – 2.36	2.47	**0.014**					
Advice condition [on-demand (No)]											0.72	0.05	0.62 – 0.83	-4.43	** < 0.001**
Advice condition [on-demand (Yes)]											1.50	0.17	1.19 – 1.88	3.47	**0.001**
Advice condition [on-demand (No)] x Prediction accuracy [incorrect]											2.71	0.60	1.76 – 4.19	4.50	** < 0.001**
Advice condition [on-demand (Yes)] x Prediction accuracy [incorrect]											0.49	0.14	0.28 – 0.84	-2.57	**0.010**
Random Effects															
σ^2^	3.29					3.29					3.29				
τ_00_	1.82 _TrialID_					1.52 _TrialID_					1.60 _TrialID_				
	0.65 _participant_					0.65 _participant_					0.65 _participant_				
ICC	0.43					0.40					0.41				
N	160 _TrialID_					160 _TrialID_					160 _TrialID_				
	80 _participant_					80 _participant_					80 _participant_				
Observations	12,800					12,800					12,800				
Marginal R^2^/Conditional R^2^	0.000/0.429					0.049/0.427					0.057/0.440				

Significant comparisons are highlighted in bold

OR > 1 is associated with higher odds for correct decision; OR < 1 is associated with lower odds for correct decision

**Fig. 3:**
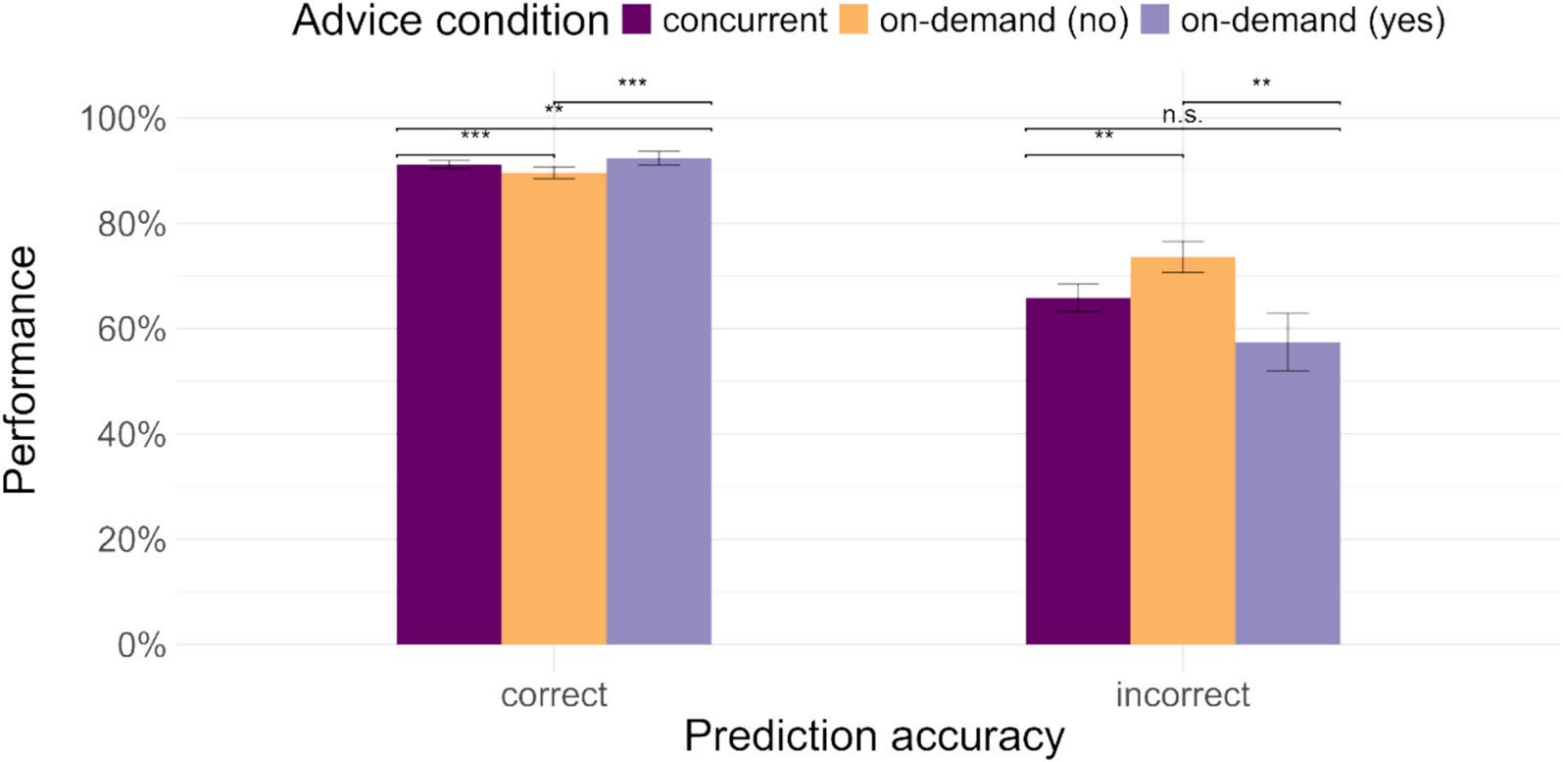
Performance on the task in the advice conditions (on-demand advice condition split by trials on which advice was demanded and not demanded) by the accuracy of the prediction

Further, we divided the trials in the on-demand advice condition into trials on which advice was demanded and trials on which no advice was demanded, and along with the concurrent advice condition, examined the impact of advice conditions and accuracy of predictions on performance (see Fig. 3). Participants demanded advice in 26.51% trials. The analysis showed that (see Table 2), on trials with correct predictions, participants performed better in the on-demand (yes) condition compared to the on-demand (no) condition, as well as the concurrent advice condition. Conversely, on trials with incorrect predictions, participants performed better in the on-demand (no) condition, compared to the other two conditions, but performance on the other two conditions was not significantly different from each other, although mean performance was lowest in the on-demand (yes) condition. These findings indicate that irrespective of whether advice is demanded or presented concurrently, correct advice helps and incorrect advice harms, though participants show a tendency to follow AI advice more when they demand it.

We then included only the trials where advice was shown (concurrently or on-demand) and examined participants’ agreement with AI advice. Overall, participants agreed with AI advice more when they demanded it than when it was shown concurrently, which was driven mainly by the correct advice cases (see eTable 2 in supplementary material). Taken together, the above findings indicate that in the on-demand advice condition, participants tend to follow advice more when they demand it; however, this is balanced by a lower agreement with predictions when advice is not demanded, which ultimately neither improves nor worsens performance compared to when advice is always presented.

## Experiment 1b

In the previous experiment, we did not find any significant improvement in performance from providing advice on-demand, as participants displayed a pattern of overreliance on advice when it was demanded. At the same time, for correct advice cases, participants made a higher number of incorrect decisions when they did not see advice compared to when they saw advice, whether it was on-demand or concurrently. Therefore, it was beneficial to see advice when it was correct, but it was better to not see advice when incorrect in order to avoid overreliance. Previous studies examined the potential of providing similarity ratings in addition to or instead of binary advice can help to reduce overreliance, but did not find any evidence for it (Carragher & Hancock, 2023; Kokje et al., 2024; Mueller et al., 2024). While such information may have value in certain instances, it is possible that this additional information may sometimes be distracting and needlessly increase complexity (Poursabzi-Sangdeh et al., 2021).

We wanted to test whether similarity ratings can overall provide more value when presented only when the user deems it necessary to see them in order to make a decision. We modified our previous paradigm to provide on-demand similarity ratings. In this experiment, binary match/mismatch advice was presented in all conditions. In addition, in the *concurrent similarity condition*, a similarity rating for each face pair was presented simultaneously by default, whereas this information was presented only when participants demanded it in the *on-demand similarity condition.*

### Participants

Eighty individuals completed the study, of which two participants were excluded for failing attention checks and one participant was excluded for completing the study too quickly (average RT < 1 s per trial). Seventy-seven participants were included in the analysis. Demographic characteristics of participants are presented in Table 3.

**Table 3:** Participant characteristics in Experiment 1b

	(*N* = 77)
*Age*	
Mean (SD)	31.8 (12.0)
*Gender*	
Female	31 (40.3%)
Male	43 (55.8%)
Non-binary	1 (1.3%)
Transgender	2 (2.6%)
*Ethnicity*	
Middle Eastern/Arab	2 (2.6%)
Turkish	1 (1.3%)
White/Caucasian	74 (96.1%)

### Stimuli and Procedure

The stimuli and procedure remained largely the same as in Experiment 1a, but we introduced similarity ratings to supplement the binary AI prediction. This means that, in the *concurrent condition,* in addition to the binary AI prediction, a similarity rating was presented for each face pair (for details on how the similarity rating was derived, refer to the supplementary material). In the *on-demand similarity condition*, face pairs were presented along with the binary AI prediction, but without the similarity ratings, which participants could choose to view if they wished by pressing a key (see Fig. 1b for example stimuli depicting advice with similarity ratings). In order to avoid any confusion about the meaning and interpretation of the similarity score, participants were provided explicit explanations that ‘the similarity score constitutes the degree of similarity between the two faces’ and ‘face pairs with a similarity score > 60% are classified as a match’.

### Results and discussion

First, we examined whether overall performance was influenced by the two advice conditions and the accuracy of the AI advice. A mixed-effects logistic regression was conducted with the advice condition and accuracy of advice as fixed factors (see eTable 4 in supplementary material). We did not find a significant effect of advice condition or an interaction between advice condition and advice accuracy. As expected, performance was significantly lower when incorrect advice was presented compared to when correct advice was presented.

**Fig. 4:**
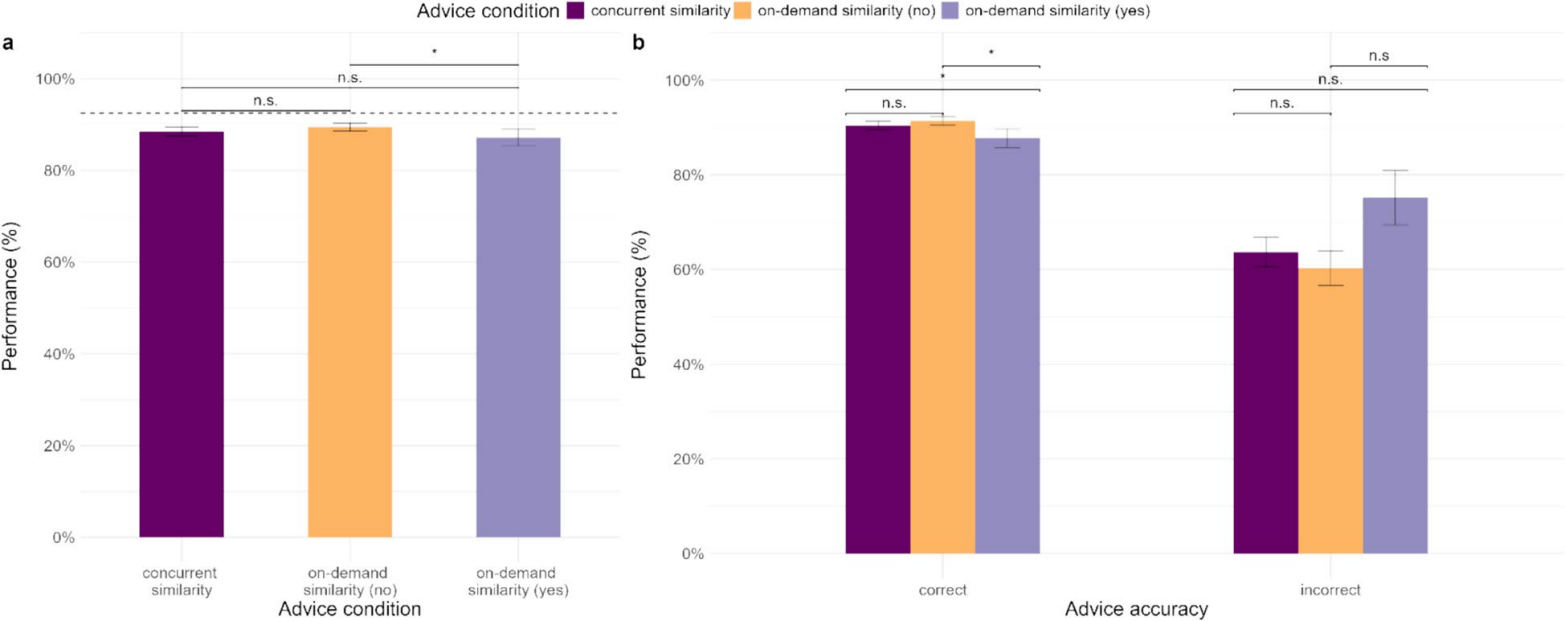
Performance on the task in (**a**) the different advice conditions (on-demand similarity condition split by trials on which similarity rating was demanded and not demanded; dotted line represents AI’s accuracy) and (**b**) the advice conditions by the accuracy of the advice

Next, we divided the trials in the on-demand similarity condition into trials where the similarity rating was demanded and trials where it was not demanded. Participants demanded similarity ratings overall in 12.6% of cases (12.0% of cases with correct advice and 19.91% of cases with incorrect advice). We explore the causes of the unequal rate of demanding similarity ratings on correct and incorrect advice trials in the supplementary material. We conducted another mixed-effects logistic regression with the three advice conditions and advice accuracy to examine performance (Table 4). Overall, participants performed worse when they demanded similarity ratings than when they did not (see Fig. 4a). Surprisingly, this effect was driven mainly by the correct advice cases (see Fig. 4b), i.e. when correct advice was presented, performance was worse when participants demanded similarity ratings compared to when they did not and when similarity ratings were presented concurrently. When incorrect advice was presented, participants’ mean performance was highest when similarity ratings were demanded, though it was not significantly better than the other two conditions.

**Table 4:** Logistic mixed-effects regression models for participants’ performance in Experiment 1b

Predictors	Performance					Performance				
	Odds Ratios	SE	95% CI	Statistic	*p*	Odds Ratios	SE	95% CI	Statistic	*p*
Intercept	17.66	2.96	12.71 – 24.54	17.11	** < 0.001**	20.94	3.43	15.19 – 28.88	18.56	** < 0.001**
on-demand [No]	1.06	0.07	0.93 – 1.21	0.90	0.366	1.05	0.08	0.91 – 1.21	0.63	0.526
on-demand [Yes]	0.73	0.10	0.56 – 0.95	-2.34	**0.020**	0.70	0.10	0.53 – 0.93	-2.45	**0.014**
Advice accuracy [incorrect]						0.09	0.04	0.04 – 0.20	-6.06	** < 0.001**
on-demand [No] x Advice accuracy [incorrect]						1.13	0.23	0.76 – 1.69	0.61	0.542
on-demand [Yes] x Advice accuracy [incorrect]						1.31	0.44	0.67 – 2.53	0.79	0.429
Random Effects										
σ^2^	3.29					3.29				
τ_00_	1.91 _TrialID_					1.49 _TrialID_				
	0.98 _participant_					0.98 _participant_				
ICC	0.47					0.43				
N	160 _TrialID_					160 _TrialID_				
	77 _participant_					77 _participant_				
Observations	12,320					12,320				
Marginal R^2^/Conditional R^2^	0.001/0.468					0.063/0.464				

Significant comparisons are highlighted in bold

We also examined agreement with AI in the three advice conditions by accuracy of advice with a mixed-effects logistic regression, which showed that participants agreed with AI less when they demanded similarity ratings, compared to when they did not and when similarity ratings were shown concurrently (see eTable 5 in the supplementary material). These results together indicate that participants likely demanded similarity ratings when they were sceptical about the AI advice and were, therefore, less likely to follow AI advice. This is noteworthy, as participants see the same correct/incorrect advice and similarity rating in the concurrent and on-demand conditions, with the only difference being whether it is presented simultaneously and by default or on-demand. From these results, it appears that participants are more trusting of AI advice that they may otherwise have been unsure about, when similarity is presented simultaneously with the advice, but a delayed presentation of similarity does not reverse scepticism about the advice. This may be beneficial when advice is incorrect, but is detrimental for correct advice.

The results from Experiments 1a and 1b together indicate that participants demand *advice* when they are indecisive, and hence, tend to agree with the advice, but they demand *similarity ratings* when they are sceptical about the advice, and hence, are less likely to follow it. We conducted a cross-experiment analysis to examine whether on-demand binary advice or on-demand similarity rating was overall more beneficial. Participants performed significantly better with binary advice, and particularly when they demanded binary advice compared to when they demanded similarity ratings (see eFig. 4 in supplementary material). Therefore, overall, similarity ratings were not just unhelpful but actually detrimental.

The above experiments provided some insight into users’ behaviour when AI advice presentation is deferred and users are given the choice of whether to view the advice. However, neither paradigm improved overall performance compared to when advice is presented concurrently and by default. Therefore, we next explore a paradigm wherein the choice of advice presentation is not up to the user, but depends on the user’s initial decision.

## Experiment 2

In this experiment, we examined whether the performance of the human–AI team may be positively influenced if AI advice is presented delayed and conditionally. Participants had to make an initial unaided decision and only received advice if their initial decision did not match the AI prediction.

### Participants

Eighty-eight individuals completed the study. Of these, we excluded two participants for completing the study too quickly (avg RT < 1 s per trial). A total of 86 participants were included in the final analysis. The demographic characteristics of our sample are presented in Table 5.

**Table 5:** Participant characteristics in Experiment 2

	(*N* = 86)
*Age*	
Mean (SD)	35.4 (9.81)
*Gender*	
Female	31 (36.0%)
Male	54 (62.8%)
Non-binary	1 (1.2%)
*Ethnicity*	
White/Caucasian	86 (100%)

### Stimuli and procedure

The experiment consisted of three advice conditions. We added a *control condition* here as the experimental advice condition involved participants making an initial unaided decision, and having the unaided control condition allowed us to directly compare potential gains in performance. In the *control condition,* participants completed the task without AI advice. In the *concurrent advice condition*, the binary AI prediction was presented along with each face pair. In the *conditional advice condition,* participants saw the face pairs without the AI prediction and were asked to register ‘match’ or ‘mismatch’ decisions *(d*_*1*_*)*. Only when the decision contradicted the AI prediction, advice was presented. Participants were then asked to register a second decision *(d*_*2*_*)*. Participants were informed that they would only be shown the AI prediction in case of a disagreement and that this did not necessarily mean that their decision was incorrect, as the AI prediction could be incorrect.

Participants were presented with 120 face pairs divided into three blocks, with 40 pairs in each block. Each block was paired with one of the three advice conditions. Three versions of the experiment were created to counterbalance the order of presentation of the conditions and to ensure that each face pair was paired with each condition. Each participant completed only one version. In the conditional advice condition, on trials when participants’ *d*_*1*_ contradicted the AI prediction, after they registered their *d*_*2*_, they were also asked to rate their confidence in their *d*_*2*_ on a scale of 1–7.

### Results and discussion

#### Performance

**Fig. 5:**
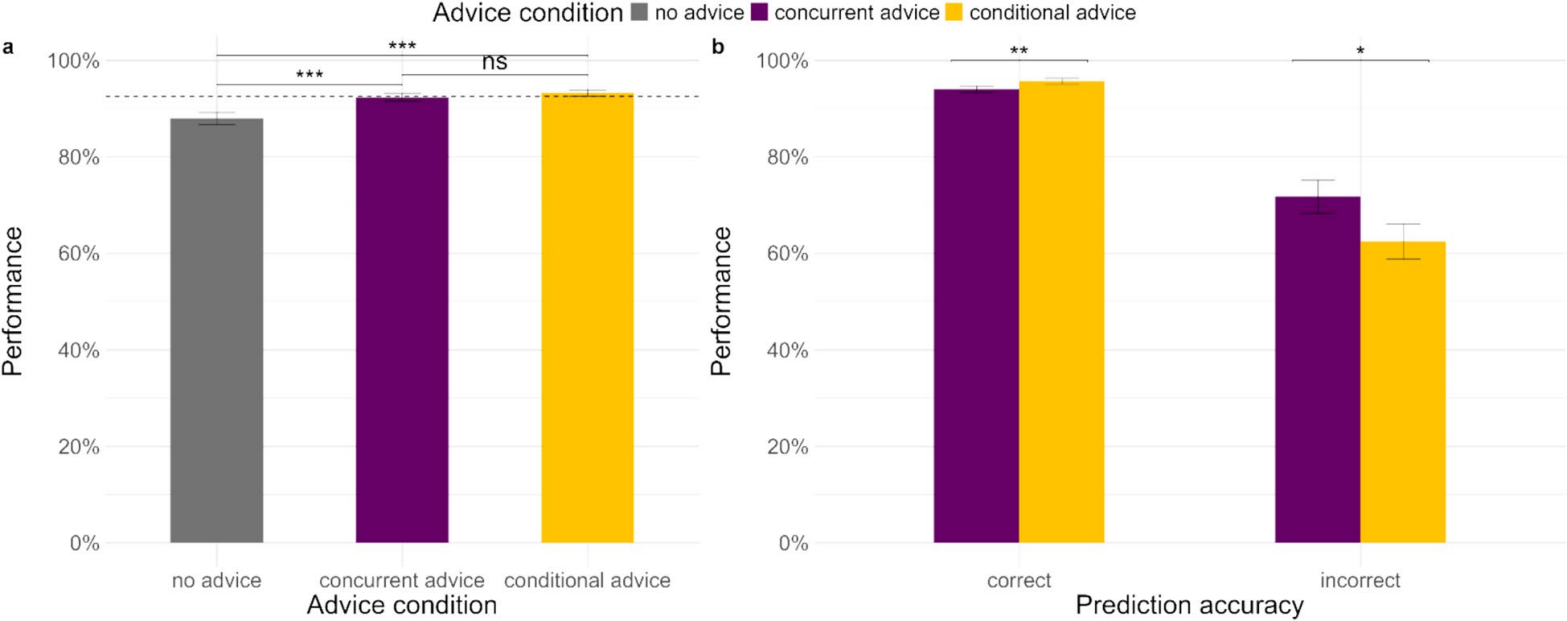
Performance on the task (**a**) overall in the different advice conditions (dotted line represents AI’s accuracy level) and (**b**) in the two conditions with advice (irrespective of whether the advice was presented or not), according to the accuracy of the prediction (see eFig. 5 in the supplementary material for performance after excluding trials where advice was not presented in the conditional advice condition)

We first examined participants’ overall performance based on the advice condition (see Fig. 5). For all analyses, unless otherwise stated, participants’ *d*_*2*_ in the conditional advice condition was included in the analysis as the final decision. Overall, when advice was presented in the conditional advice condition, participants switched their decision in 43.58% of cases. A mixed-effects logistic regression (see Table 6) showed that, overall, participants performed significantly better in both conditions where they received advice compared to the no advice condition. Their performance in the two conditions with advice did not differ significantly. However, notably, for the first time in our experiments, the mean performance in the conditional advice condition (93.17%) was better than the AI alone (92.5%), albeit only marginally.

**Table 6:** Logistic mixed-effects regression models for participants’ performance in Experiment 2

Predictors	Performance					Performance				
	Odds Ratios	SE	95% CI	Statistic	*p*	Odds Ratios	SE	95% CI	Statistic	*p*
Intercept	15.43	2.64	11.03 – 21.58	15.98	** < 0.001**	36.71	7.01	25.25 – 53.38	18.86	** < 0.001**
Condition [Concurrent advice]	1.87	0.17	1.56 – 2.24	6.80	** < 0.001**					
Condition [Conditional advice]	2.19	0.21	1.81 – 2.63	8.23	** < 0.001**	1.48	0.18	1.16 – 1.88	3.15	**0.002**
Prediction accuracy [incorrect]						0.09	0.04	0.03 – 0.23	-4.94	** < 0.001**
Condition [Conditional advice] x Prediction accuracy [incorrect]						0.36	0.10	0.21 – 0.62	-3.71	** < 0.001**
Random Effects										
σ^2^	3.29					3.29				
τ_00_	1.82 _TrialID_					1.55 _TrialID_				
	0.75 _participant_					0.76 _participant_				
ICC	0.44					0.41				
N	120 _TrialID_					120 _TrialID_				
	86 _participant_					86 _participant_				
Observations	10,320					6880				
Marginal R^2^/Conditional R^2^	0.019/0.450					0.101/0.473				

Significant comparisons are highlighted in bold

OR > 1 is associated with higher odds for correct decision; OR < 1 is associated with lower odds for correct decision

We also examined the effect of the interaction between advice condition and accuracy of the AI prediction on performance via a mixed-effects logistic regression (Table 6). We include all trials in both conditions including trials in the conditional advice condition where no advice was presented. We found that performance was significantly better in the conditional advice condition on trials with correct AI predictions, whereas when AI predictions were incorrect, performance was significantly better in the concurrent advice condition (Fig. 5b). The lower performance in the conditional advice condition for incorrect prediction cases appears to be driven by cases where participants’ d_1_ is incorrect, but it matches the incorrect AI prediction, thereby never triggering a contradiction. When we exclude these cases, performance is better in the conditional compared to concurrent advice condition (see eFig. 5 in supplementary material).

#### AI agreement

**Fig. 6:**
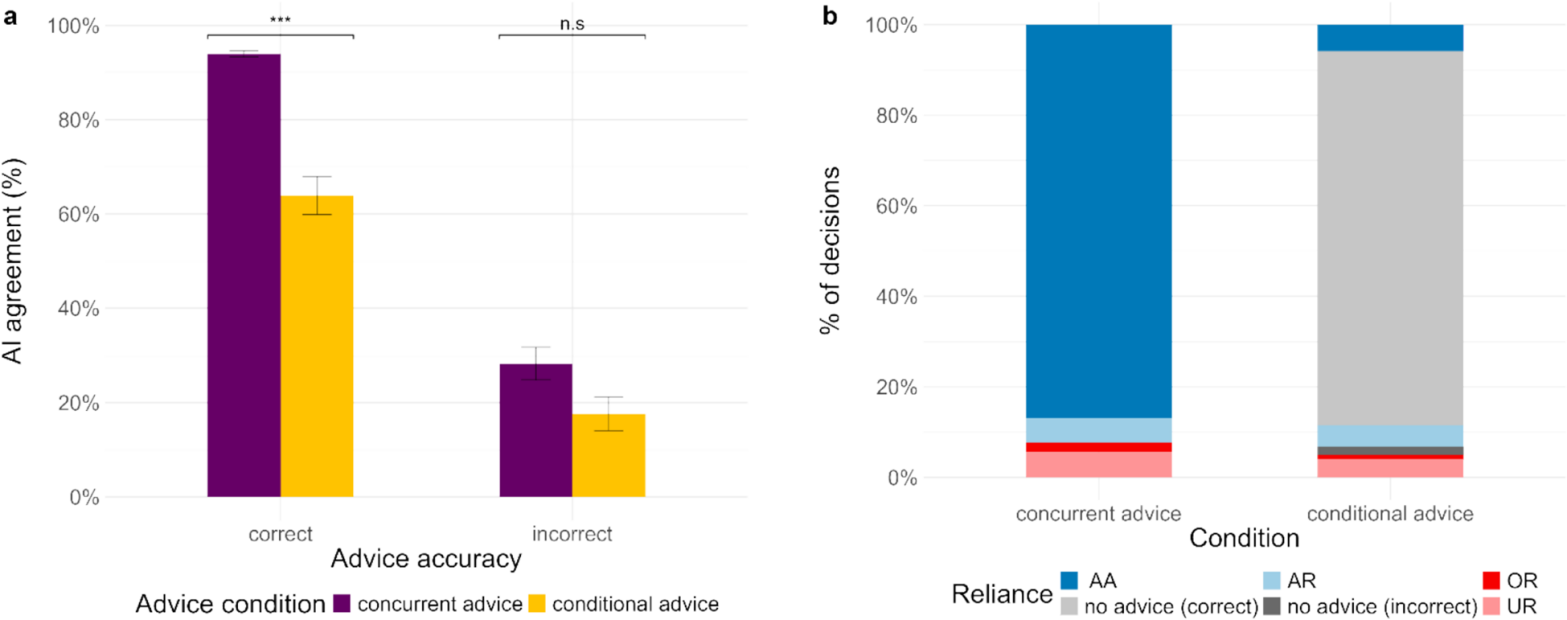
AA = appropriate acceptance, AR = appropriate rejection, OR = overreliance, UR = underreliance. (**a**) Percentage of decisions in agreement with the AI advice, when AI advice was presented and (**b**) Percentage of decisions according to the type of reliance on AI advice

To examine participants’ response pattern on trials where AI advice was presented, we look at AI agreement. We conducted a mixed-effects logistic regression with advice condition and advice accuracy as fixed factors (see eTable 6). Participants’ agreement with the AI advice was significantly higher in the concurrent advice condition compared to the conditional advice condition in the correct advice cases (see Fig. 6a). In incorrect advice cases, the difference was not significant, possibly because of the lack of power due to the low number of cases. However, when we account for the cases where participants made the same d_1_ as the AI prediction (89.38% for correct and 25.58% for incorrect advice cases), agreement with the AI prediction was actually higher in the conditional advice condition.

Further, we were interested in examining participants’ reliance patterns on AI advice in the two advice conditions. We classified participants’ responses as the type of reliance on advice—appropriate acceptance (participants accepted correct advice), appropriate rejection (participants rejected incorrect advice), overreliance (participants accepted incorrect advice), and underreliance (participants rejected correct advice). Additionally, in the conditional advice condition, the proportion of responses where participants did not see any advice (as their first response was in agreement with AI prediction) is also calculated as *no advice (correct)* or *no advice (incorrect)* depending on whether their response and the AI prediction were correct or incorrect. The breakdown of the proportion of each type of decision in the two advice conditions is provided in Fig. 6b. Participants’ underreliance on correct advice (5.58% vs. 4.01%) and overreliance on incorrect advice (2.12% vs. 0.9%) were both significantly lower in the conditional advice condition. However, in 1.92% cases participants’ d_1_ was the same as the incorrect AI prediction, which ultimately resulted in an overall higher number of cases (2.82%) where participants’ decision was in agreement with incorrect AI predictions.

#### Confidence

**Fig. 7:**
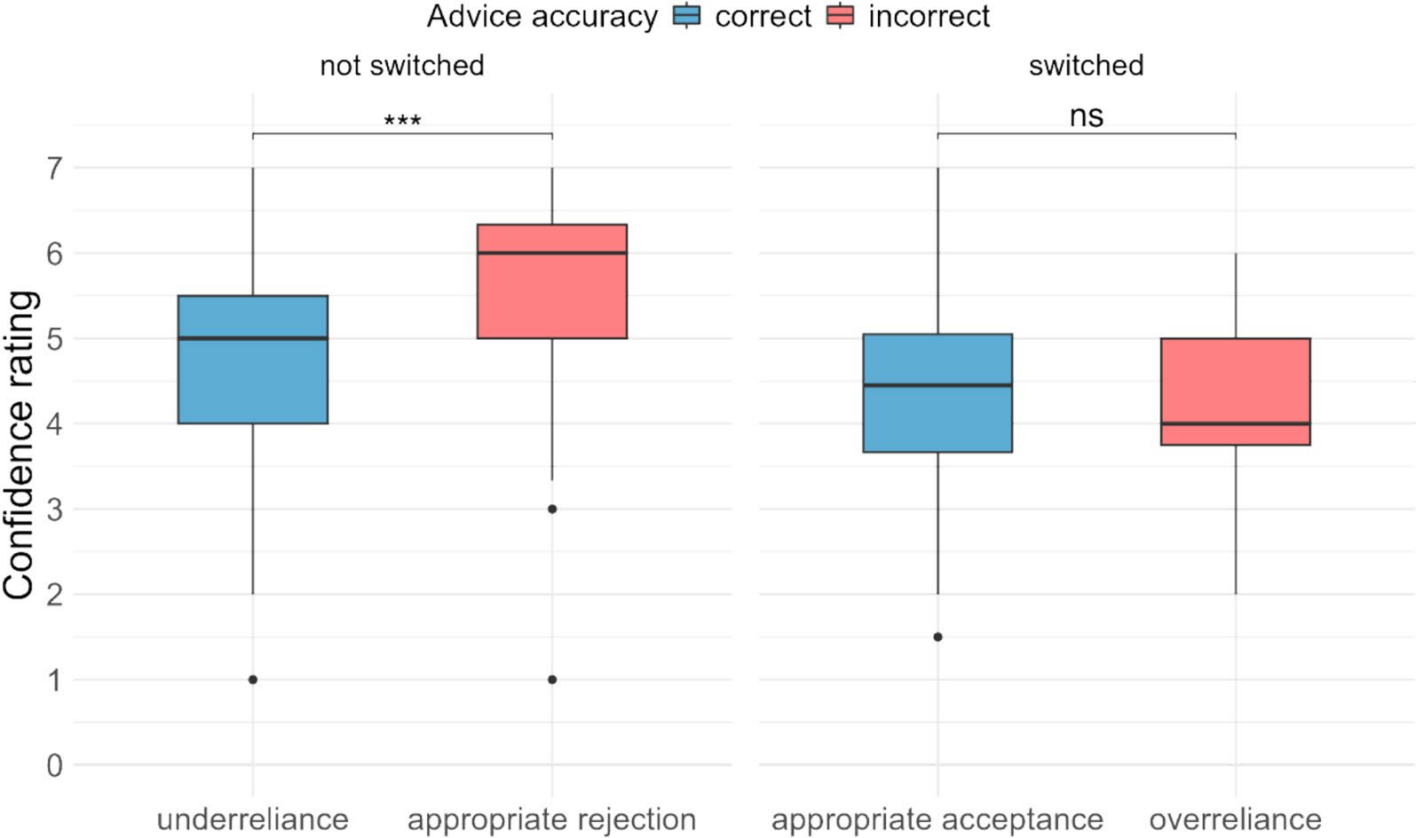
Participants’ confidence in their decision in the conditional advice condition on trials where AI advice was presented by whether they switched their initial decision or not and accuracy of the advice

In the conditional advice condition, on trials where AI advice was presented, participants were asked to rate their confidence in their second decision. We examined confidence based on whether participants switched their decision from the initial decision, and whether the AI advice was correct or incorrect (see Fig. 7). We conducted an ordinal mixed-effects regression with switch condition and advice accuracy as fixed factors (see eTable 7). Participants were more confident in their decision when they were rejecting incorrect advice (i.e. appropriate rejection) compared to both types of error responses—underreliance and overreliance—indicating that participants experience increased uncertainty in their decision when making errors. Notably, when participants did change their incorrect d_1_ in response to correct advice (i.e. appropriate acceptance), they were not more confident than when they made error responses despite making the correct decision. Together, these results indicate that participants view AI advice with considerable scepticism when they have had the opportunity to form their own judgement first and are presented with contradictory advice. Therefore, they feel more confident rejecting incorrect advice, but not as confident accepting correct advice.

### Confidence

## General discussion

The aim of this study was to examine the potential of non-concurrent AI advice presentation strategies to mitigate overreliance on AI advice. We investigated on-demand advice, on-demand similarity rating, and conditional advice based on the incongruency of user decisions with the AI prediction—all conditions require the user to engage with the task before they are exposed to AI advice. Although we did not find significant improvement in overall performance with any of the delayed presentation strategies compared to concurrent advice presentation, we did find noteworthy response patterns.

First, we found that participants were more likely to follow AI advice when they demanded it compared to when it was presented concurrently, irrespective of whether the advice was correct or incorrect. This indicates that uncertainty in their decision leads users to demand advice, and thus, overreliance does not decrease when users demand advice as they are unsure. However, when participants did not demand advice on trials with incorrect AI predictions, they performed better than both conditions where they saw the AI advice (concurrently or on-demand). But, they also made more errors on trials with correct AI predictions when they did not demand advice compared to when they saw advice. Therefore, overall performance in the on-demand advice condition neither significantly improved nor declined compared to the concurrent advice condition. It is also noteworthy that participants demanded advice, on an average, on 26.51% of trials, which is considerably low, so, overall reliance on AI was lower in the on-demand condition.

Second, participants followed AI advice less frequently when they demanded the similarity rating irrespective of whether the advice was correct or incorrect. This indicates that users demanded ratings when they were sceptical about the binary advice. This harmed performance when advice was correct, but improved performance for incorrect advice case. However, the latter effect was not significant, likely due to a lack of power due to the small number of incorrect advice cases. Our finding that demanding similarity ratings decreased reliance on AI contradict the findings from Papenkordt (2024), wherein demanding explanations increased overall reliance on AI advice, thereby reducing underreliance on correct advice but not overreliance on incorrect advice. Buçinca et al. (2021), on the other hand, found that on-demand explanations, among other alternate presentation strategies, reduced overreliance compared to concurrent explanations. However, it is difficult to determine whether this was specifically the case for the on-demand condition, as they report results for the on-demand condition combined with other presentation strategies. It is important to note here that the task and type of additional information provided in these studies was different from our study. Bucinca et. al employed a task on nutritional choices and Papenkordt employed an object-classification task. Both studies used descriptive textual, feature-based explanations, and Papenkordt additionally employed a numerical explanation in the form of certainty of the recommendation. While the textual explanations in the other studies provided a rationale for the AI predictions, the output generated by the algorithm in our study was not as explanatory in nature, as it was a single numerical value with no additional rationale for it.

Overall, we did not find any benefit in providing similarity ratings, concurrently or on-demand, over binary advice, in improving performance. Previous studies have suggested that explanation complexity contributes significantly to the utility of explanations, where simpler explanations are useful, but more complex explanations may increase overreliance (Cecil et al., 2024; Schemmer et al., 2022; Westphal et al., 2023). In the face recognition literature, richer forms of explanation have been proposed, such as visual saliency and feature attribution maps, or textual explanations describing similarities/differences, that indicate what facial regions or features were most influential in the AI’s decision, rather than only a summary similarity score (Boyd et al., 2022; Doh et al., 2025; Williford et al., 2020). These methods also increase complexity and cognitive load for users, and user studies implementing these forms of explanations have been lacking. The similarity ratings in our study were simple to understand but did not really explain the decision, and it is likely that participants simply did not find the information elaborate enough to provide additional assistance in making their decision. However, one study (Boyd et al., 2022), that implemented visual saliency maps as an explanation in a synthetic face identification task, also reported an improvement in performance with a binary AI decision, but not with the presentation of visual saliency maps. The on-demand similarity ratings paradigm compared to concurrent presentation at least shows some promise for reducing overreliance on incorrect advice, as the lack of improvement in performance here resulted from underreliance on AI advice, whereas studies that report adverse effects of explainable advice widely attribute it to increased overreliance due to increased trust in AI (Bertrand et al., 2022; Rosenbacke et al., 2024; Schemmer et al., 2022).

Finally, in Experiment 2, participants followed AI advice less when it was only presented following a contradiction between the AI prediction and their own judgement (i.e. conditional advice condition). This is also reflected in the confidence ratings, wherein participants were more confident rejecting incorrect AI advice than they were when accepting correct AI advice, indicating a lower automation bias in this condition. This effect appears to be beneficial, as the proportion of incorrect advice cases where a contradiction was triggered was high (74.42%) and of these, cases where participants did not switch their initial (correct) decision was high too (83.85%). Conversely, for correct advice cases the proportion where they did not switch their (incorrect) decision is relatively lower (40.83%). The number of contradictions was also relatively low for the correct advice cases (10.62%), and therefore, performance was better in the conditional advice condition for correct advice cases. While the confidence ratings might also suggest a reduced anchoring bias in the conditional advice condition, we cannot confirm this as we did not measure confidence in both conditions, and thus cannot make a direct comparison of confidence in the two advice conditions. Further studies employing this paradigm in a non-binary decision-making task may be useful to examine this. In our study, although overall performance was not significantly different in the advice conditions, it is noteworthy that the average group performance (93.17%) marginally exceeded the accuracy rate of the AI alone (92.5%) in the conditional advice condition.

Overall, none of our paradigms resulted in better overall performance, but participants tended to follow AI advice less in the non-concurrent advice conditions, indicating that delaying advice presentation reduces automation bias and compels the user to engage with the decision even if they ultimately choose to see the AI advice. The study also highlights that users are more reluctant to accept AI advice once they have decided and are less confident in the decision if they choose to accept contradictory AI advice. So, these paradigms have potential in terms of reducing overreliance, but at the cost of higher underreliance. Therefore, whether concurrent or non-concurrent advice presentation has greater utility ultimately depends on the context or task at hand. In high-risk contexts, like passport control or healthcare, a relatively higher degree of scepticism would be preferred over a higher automation bias. In such cases, delayed advice presentation methods may have more utility. On the other hand, in low-risk contexts where saving time may be more essential, concurrent advice paradigms may prove to be more useful. Another aspect to consider is the accuracy rate of the algorithm. With high-accuracy algorithms that massively outperform humans, as tends to be the case with face matching, underreliance on correct advice would be a bigger concern than overreliance on incorrect advice. It is also possible that depending on the use case, users may actually interact differently with the advice paradigms in this study. Our study was conducted with non-experts in a low-risk context, though we did incentivize performance to raise the stakes. Further studies that employ these paradigms in different use cases of varying risk levels are required to evaluate the generalizability of these findings.

## Limitations

Our study was not conducted specifically with experts, i.e. people who may perform facial identity verification as part of their job. It is possible that users who perform such a task daily may interact differently with the AI advice. Although it has been established that performing facial identification regularly or even receiving training, does not improve one’s ability (White et al., 2014), some beneficial effects of experience with using an AI aid in the face-matching context regularly cannot be ruled out.

In Experiment 2, we included a confidence measure. However, for the sake of time and limiting monotony, we did not implement this measure in all conditions and on all trials, but only in the conditional advice condition when the AI advice was triggered. Although the measure was rather helpful in understanding participants’ behaviour when a contradiction arises, it also precluded us from a direct comparison with when there is no contradiction, as well as, when advice is presented concurrently. Therefore, we cannot determine whether participants would feel equally confident rejecting incorrect advice when it is presented concurrently as they did when following a contradiction.

In order to have an experimental paradigm that is closer to real-world conditions, we used an actual AI model to generate predictions that we used in the study. As we tried to maintain the true accuracy rate of the AI model in our experiments, the number of incorrect predictions was low. As a result of this, the study may have sometimes lacked power for the incorrect advice cases.

## Conclusion

The study proposed paradigms involving non-concurrent AI advice presentation as a potential solution to the problem of overreliance on AI in human–AI collaboration. While our findings do indicate that there is potential to reduce overreliance with these strategies, it also increases the possibility of underreliance, either due to actively rejecting correct advice or due to never seeing advice at all. As a result, overall performance neither improved nor declined in comparison with concurrent default advice presentation. Therefore, we cannot uniformly recommend one or the other paradigm, but instead conclude that the varying reliance patterns in the two types of paradigms should be taken into account while deciding which paradigm may be more appropriate for the specific use case and context in which the AI system is being implemented. We also acknowledge that these findings may not be generalizable to all contexts and recommend that further studies should test the paradigms in different fields.

## Supplementary Information

Additional file1 (DOCX 1444 KB)

## Abbreviations

AI: Artificial Intelligence
AI-DSS: AI-based Decision Support Systems
GUFD: Glasgow University Face Database

## References

[CR1] Agarwal, N., Huang, R., Moehring, A., Rajpurkar, P., Salz, T., & Yu, F. (2024). Comparative advantage of humans versus AI in the long tail. *AEA Papers and Proceedings,**114*, 618–622. 10.1257/pandp.20241071

[CR2] Bertrand, A., Belloum, R., Eagan, J. R., & Maxwell, W. (2022). How Cognitive Biases Affect XAI-assisted Decision-making: A Systematic Review. *Proceedings of the 2022 AAAI/ACM Conference on AI, Ethics, and Society* (pp. 78–91). 10.1145/3514094.3534164

[CR3] Bertrand, A., Eagan, J. R., & Maxwell, W. (2023). Questioning the ability of feature-based explanations to empower non-experts in robo-advised financial decision-making. *Proceedings of the 2023 ACM Conference on Fairness, Accountability, and Transparency* (pp. 943–958). 10.1145/3593013.3594053

[CR4] Boyd, A., Tinsley, P., Bowyer, K., & Czajka, A. (2022). The value of AI guidance in human examination of synthetically-generated faces. *Proceedings of the AAAI Conference on Artificial Intelligence,**37*(5), 5930–5938. 10.1609/aaai.v37i5.25734

[CR5] Buçinca, Z., Malaya, M. B., & Gajos, K. Z. (2021). To trust or to think: Cognitive forcing functions can reduce overreliance on AI in AI-assisted decision-making. *Proceedings of the ACM on Human-Computer Interaction,**5*(CSCW1), 1–21. 10.1145/344928736644216

[CR6] Burton, A. M., White, D., & McNeill, A. (2010). The Glasgow Face Matching Test. *Behavior Research Methods,**42*(1), 286–291. 10.3758/BRM.42.1.286 20160307 10.3758/BRM.42.1.286

[CR7] Carragher, D. J., & Hancock, P. J. (2023). Simulated automated facial recognition systems as decision-aids in forensic face matching tasks. *Journal of Experimental Psychology: General,**152*(5), 1286. 10.1037/xge0001310 36455036 10.1037/xge0001310

[CR8] Carragher, D. J., Sturman, D., & Hancock, P. J. B. (2024). Trust in automation and the accuracy of human–algorithm teams performing one-to-one face matching tasks. *Cognitive Research: Principles and Implications,**9*(1), Article 41. 10.1186/s41235-024-00564-8 38902539 10.1186/s41235-024-00564-8 PMC11190114

[CR9] Cecil, J., Lermer, E., Hudecek, M. F. C., Sauer, J., & Gaube, S. (2024). Explainability does not mitigate the negative impact of incorrect AI advice in a personnel selection task. *Scientific Reports,**14*(1), Article 9736. 10.1038/s41598-024-60220-5 38679619 10.1038/s41598-024-60220-5 PMC11056364

[CR10] Choi, J. H., & Schwarcz, D. (2023). AI assistance in legal analysis: An empirical study. *Journal of Legal Education*. 10.2139/ssrn.4539836

[CR11] Cohen, M. C., Mancenido, M. V., Chiou, E. K., & Cooke, N. J. (2023). *Teamness and Trust in AI-Enabled Decision Support Systems: Current Challenges and Future Directions*. (*3456*, 175–187).

[CR12] Doh, M., Rodrigues, C. M., Boutry, N., Najman, L., Mancas, M., & Gosselin, B. (2025). Found in Translation: Semantic approaches for enhancing AI interpretability in face verification. *ArXiv Preprint ArXiv:2501.05471*, 10.48550/arXiv.2501.05471

[CR13] Fogliato, R., Chappidi, S., Lungren, M., Fisher, P., Wilson, D., Fitzke, M., Parkinson, M., Horvitz, E., Inkpen, K., & Nushi, B. (2022). Who Goes First? Influences of Human-AI Workflow on Decision Making in Clinical Imaging. *Proceedings of the 2022 ACM Conference on Fairness, Accountability, and Transparency* (pp. 1362–1374). 10.1145/3531146.3533193

[CR14] Furnham, A., & Boo, H. C. (2011). A literature review of the anchoring effect. *The Journal of Socio-Economics,**40*(1), 35–42.10.1016/j.socec.2010.10.008

[CR15] Fysh, M. C., & Bindemann, M. (2018). Human–computer interaction in face matching. *Cognitive Science,**42*(5), 1714–1732. 10.1111/cogs.12633 29954047 10.1111/cogs.12633 PMC6099365

[CR16] Gajos, K. Z., & Mamykina, L. (2022). Do People Engage Cognitively with AI? Impact of AI Assistance on Incidental Learning. *Proceedings of the 27th International Conference on Intelligent User Interfaces* (pp. 794–806). 10.1145/3490099.3511138

[CR17] Gaube, S., Jussupow, E., Kokje, E., Khan, J., Bondi-Kelly, E., & Schicho, A. (2024). Underreliance Harms Human-AI Collaboration More Than Overreliance in Medical Imaging. 10.31219/osf.io/4wv8j

[CR18] Goddard, K., Roudsari, A., & Wyatt, J. (2011). Decision support and automation bias: Methodology and preliminary results of a systematic review. *International Perspectives in Health Informatics*, 3–7. 21335679

[CR19] Grother, P., Ngan, M., Hanaoka, K., Yang, J. C., & Hom, A. (2024). Face Recognition Technology Evaluation (FRTE) Part 1: Verification. *Technical Report, National Institute of Standards and Technology,**09*, 2023.

[CR20] Gupta, S., Modgil, S., Lee, C.-K., & Sivarajah, U. (2023). The future is yesterday: Use of AI-driven facial recognition to enhance value in the travel and tourism industry. *Information Systems Frontiers,**25*(3), 1179–1195. 10.1007/s10796-022-10271-8 35529102 10.1007/s10796-022-10271-8 PMC9059456

[CR21] Hancock, P. J., Somai, R. S., & Mileva, V. R. (2020). Convolutional neural net face recognition works in non-human-like ways. *Royal Society Open Science,**7*(10), Article 200595. 10.1098/rsos.200595 33204449 10.1098/rsos.200595 PMC7657890

[CR22] Higgins, O., Short, B. L., Chalup, S. K., & Wilson, R. L. (2023). Artificial intelligence (AI) and machine learning (ML) based decision support systems in mental health: An integrative review. *International Journal of Mental Health Nursing,**32*(4), 966–978. 10.1111/inm.13114 36744684 10.1111/inm.13114

[CR23] Howard, J. J., Rabbitt, L. R., & Sirotin, Y. B. (2020). Human-algorithm teaming in face recognition: How algorithm outcomes cognitively bias human decision-making. *PLoS ONE,**15*(8), Article e0237855. 10.1371/journal.pone.0237855 32822441 10.1371/journal.pone.0237855 PMC7444527

[CR24] Jacobs, M., Pradier, M. F., McCoy, T. H., Perlis, R. H., Doshi-Velez, F., & Gajos, K. Z. (2021). How machine-learning recommendations influence clinician treatment selections: The example of antidepressant selection. *Translational Psychiatry,**11*(1), Article 108. 10.1038/s41398-021-01224-x 33542191 10.1038/s41398-021-01224-x PMC7862671

[CR25] Jansen, M., Nguyen, H. Q., & Shams, A. (2024). Rise of the machines: The impact of automated underwriting. *Management Science*. 10.1287/mnsc.2024.4986

[CR26] Klingbeil, A., Grützner, C., & Schreck, P. (2024). Trust and reliance on AI — An experimental study on the extent and costs of overreliance on AI. *Computers in Human Behavior,**160*, Article 108352. 10.1016/j.chb.2024.108352

[CR27] Kokje, E., Lermer, E., Donkin, C., & Gaube, S. (2024). Understanding the influence of design-related factors on human-AI teaming in a face matching task. *Cognitive Research: Principles and Implications,**11*(1), 4. 10.1186/s41235-025-00701-x 10.1186/s41235-025-00701-x PMC12779867 41501590

[CR28] Kumar, A., Patel, T., Benjamin, A. S., & Steyvers, M. (2021). *Explaining algorithm aversion with metacognitive bandits*. *43*(43).

[CR29] Liang, G., Sloane, J. F., Donkin, C., & Newell, B. R. (2022). Adapting to the algorithm: How accuracy comparisons promote the use of a decision aid. *Cognitive Research: Principles and Implications,**7*(1), Article 14. 10.1186/s41235-022-00364-y 35133521 10.1186/s41235-022-00364-y PMC8825899

[CR30] Megreya, A. M., & Burton, A. M. (2008). Matching faces to photographs: Poor performance in eyewitness memory (without the memory). *Journal of Experimental Psychology: Applied,**14*(4), 364–372. 10.1037/a0013464 19102619 10.1037/a0013464

[CR31] Mueller, M., Hancock, P. J. B., Cunningham, E. K., Watt, R. J., Carragher, D., & Bobak, A. K. (2024). Automated face recognition assists with low-prevalence face identity mismatches but can bias users. *British Journal of Psychology*. 10.1111/bjop.12745 39545786 10.1111/bjop.12745 PMC13051006

[CR32] O’Toole, A. J., Abdi, H., Jiang, F., & Phillips, P. J. (2007). Fusing face-verification algorithms and humans. *IEEE Transactions on Systems, Man, and Cybernetics, Part B (Cybernetics),**37*(5), 1149–1155. 10.1109/TSMCB.2007.907034 10.1109/tsmcb.2007.907034 17926698

[CR33] Papenkordt, J. (2024). Navigating Transparency: The Influence of On-demand Explanations on Non-expert User Interaction with AI. In H. Degen & S. Ntoa (Eds.), *Artificial Intelligence in HCI* (pp. 238–263). Springer Nature Switzerland.

[CR34] Park, J. S., Barber, R., Kirlik, A., & Karahalios, K. (2019). A slow algorithm improves users’ assessments of the algorithm’s accuracy. *ACM Hum.-Comput. Interact Proc*. 10.1145/3359204

[CR35] Passi, S., & Vorvoreanu, M. (2022). Overreliance on AI literature review. *Microsoft Research.,**339*, 340.

[CR36] Peirce, J. W. (2007). PsychoPy—Psychophysics software in Python. *Journal of Neuroscience Methods,**162*(1), 8–13. 10.1016/j.jneumeth.2006.11.017 17254636 10.1016/j.jneumeth.2006.11.017 PMC2018741

[CR37] Phillips, P. J., Yates, A. N., Hu, Y., Hahn, C. A., Noyes, E., Jackson, K., Cavazos, J. G., Jeckeln, G., Ranjan, R., Sankaranarayanan, S., Chen, J.-C., Castillo, C. D., Chellappa, R., White, D., & O’Toole, A. J. (2018). Face recognition accuracy of forensic examiners, superrecognizers, and face recognition algorithms. *Proceedings of the National Academy of Sciences,**115*(24), 6171–6176. 10.1073/pnas.1721355115 10.1073/pnas.1721355115 PMC6004481 29844174

[CR38] Poursabzi-Sangdeh, F., Goldstein, D. G., Hofman, J. M., Wortman Vaughan, J. W., & Wallach, H. (2021). Manipulating and measuring model interpretability. 1–52.

[CR39] Rajpurkar, P., Chen, E., Banerjee, O., & Topol, E. J. (2022). AI in health and medicine. *Nature Medicine,**28*(1), 31–38. 10.1038/s41591-021-01614-0 35058619 10.1038/s41591-021-01614-0

[CR40] Rosenbacke, R., Melhus, Å., McKee, M., & Stuckler, D. (2024). How explainable artificial intelligence can increase or decrease clinicians’ trust in AI applications in health care: Systematic review. *JMIR AI,**3*, Article e53207. 10.2196/53207 39476365 10.2196/53207 PMC11561425

[CR41] Schemmer, M., Kühl, N., Benz, C., & Satzger, G. (2022). On the influence of explainable AI on automation bias. *ArXiv Preprint ArXiv:2204.08859*, 10.48550/arXiv.2204.08859

[CR42] Schroff, F., Kalenichenko, D., & Philbin, J. (2015). FaceNet: A unified embedding for face recognition and clustering. *IEEE Conference on Computer Vision and Pattern Recognition (CVPR),**2015*, 815–823.10.1109/CVPR.2015.7298682

[CR43] Senoner, J., Schallmoser, S., Kratzwald, B., Feuerriegel, S., & Netland, T. (2024). Explainable AI improves task performance in human–AI collaboration. *Scientific Reports,**14*(1), Article 31150. 10.1038/s41598-024-82501-9 39730794 10.1038/s41598-024-82501-9 PMC11681242

[CR44] Skitka, L. J., Mosier, K. L., & Burdick, M. (1999). Does automation bias decision-making? *International Journal of Human-Computer Studies,**51*(5), 991–1006.

[CR45] Steyvers, M., & Kumar, A. (2024). Three challenges for AI-assisted decision-making. *Perspectives on Psychological Science,**19*(5), 722–734. 10.1177/17456916231181102 37439761 10.1177/17456916231181102 PMC11373149

[CR46] Vasconcelos, H., Jörke, M., Grunde-McLaughlin, M., Gerstenberg, T., Bernstein, M. S., & Krishna, R. (2023). Explanations can reduce overreliance on AI systems during decision-making. *Proceedings of the ACM on Human-Computer Interaction,**7*(CSCW1), 1–38. 10.1145/3579605

[CR47] Westphal, M., Vössing, M., Satzger, G., Yom-Tov, G. B., & Rafaeli, A. (2023). Decision control and explanations in human-AI collaboration: Improving user perceptions and compliance. *Computers in Human Behavior,**144*, Article 107714. 10.1016/j.chb.2023.107714

[CR48] White, D., Kemp, R. I., Jenkins, R., Matheson, M., & Burton, A. M. (2014). Passport officers’ errors in face matching. *PLoS ONE,**9*(8), Article e103510. 10.1371/journal.pone.0103510 25133682 10.1371/journal.pone.0103510 PMC4136722

[CR49] Williford, J. R., May, B. B., & Byrne, J. (2020). Explainable face recognition. 248–263.

[CR50] Yang, Q., Steinfeld, A., Rosé, C., & Zimmerman, J. (2020). Re-examining Whether, Why, and How Human-AI Interaction Is Uniquely Difficult to Design. *Proceedings of the 2020 CHI Conference on Human Factors in Computing Systems* (pp. 1–13). 10.1145/3313831.3376301

[CR51] Yoon, J. H., Strand, F., Baltzer, P. A. T., Conant, E. F., Gilbert, F. J., Lehman, C. D., Morris, E. A., Mullen, L. A., Nishikawa, R. M., Sharma, N., Vejborg, I., Moy, L., & Mann, R. M. (2023). Standalone AI for breast cancer detection at screening digital mammography and digital breast tomosynthesis: A systematic review and meta-analysis. *Radiology,**307*(5), Article e222639. 10.1148/radiol.222639 37219445 10.1148/radiol.222639 PMC10315526

[CR52] Yousaf, A., Kayvanfar, V., Mazzoni, A., & Elomri, A. (2023). Artificial intelligence-based decision support systems in smart agriculture: Bibliometric analysis for operational insights and future directions. *Frontiers in Sustainable Food Systems*. 10.3389/fsufs.2022.1053921

[CR53] Zhang, Z. T., Buchner, F., Liu, Y., & Butz, A. (2024). You can only verify when you know the answer: feature-based explanations reduce overreliance on AI for easy decisions, but not for hard ones. *Proceedings of Mensch und Computer,**2024*, 156–170. 10.1145/3670653.3670660

[CR54] Zhang, Y., Liao, Q. V., & Bellamy, R. K. E. (2020). Effect of confidence and explanation on accuracy and trust calibration in AI-assisted decision making. *Proceedings of the 2020 Conference on Fairness, Accountability, and Transparency* (pp. 295–305). 10.1145/3351095.3372852

