# Supplementary material for: AI-augmented decision-making in face matching: comparing concurrent and non-concurrent advice presentation
Source: Cogn Res Princ Implic. 2026 Feb 5;11:11. doi: 10.1186/s41235-026-00707-z (PMC12876487; doi:10.1186/s41235-026-00707-z)
Supplement: Supplementary file 1 — Additional file1 (DOCX 1444 KB) [file 41235_2026_707_MOESM1_ESM.docx]

# Online Supplements

# Supplementary Methods: Stimuli

## AI predictions

For generating the AI predictions, We used an openly available deep convolutional neural network (DCNN) based AI model called Facenet (Schroff et al., 2015), within deepface (<https://github.com/serengil/deepface>), which is a face recognition and facial attribute analysis framework for Python. We ran 100 face pairs from each database (GUFD and the Arab face set) through the model, which generated a ‘distance’ value, i.e., the degree of dissimilarity between the two faces, which ranges from 0 to 1, with higher values indicating greater distance. The default metric in the framework, cosine similarity, was used to compute this distance. Each face was represented as a 128-dimensional embedding vector. The similarity between two embeddings was computed to get the cosine distance (1- cosine similarity). Based on this distance value, a binary prediction of ‘match’ or ‘mismatch’ was generated. Facenet uses a threshold value of .40. Face pairs with a distance value > .40 are classified as mismatches, and lower than this are classified as matches. Of the 200 face pairs, the model’s predictions were accurate in 92.9% cases. We used the actual predictions generated and attempted to maintain approximately the true accuracy rate in the experiments.

In order to evaluate whether the default threshold (0.40) for classifying matches and mismatches, was appropriate for the current dataset, we examined the distance distributions for the match and mismatch pairs. As seen in eFigure 1, the two distributions are well separated, with match pairs clustering around smaller distances (*M* = 0.18, *SD* = 0.13), while mismatch pairs clustering around larger distances (*M* = 0.64, *SD* = 0.16). The default threshold falls between these distributions, indicating that it provides an appropriate threshold for this dataset.


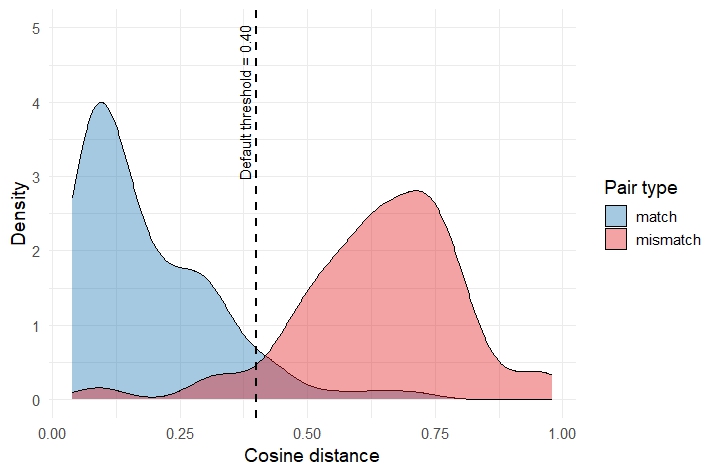


*eFigure 1*: Distribution of cosine distance scores for match and mismatch face pairs.

# Supplementary Results

# *eTable 1*: Means and SDs in the different conditions in Experiments 1a, 1b, and 2.

| **Experiment 1a** | | |  |  | | |  | |  | | |  | |  | |  |  |  |
| --- | --- | --- | --- | --- | --- | --- | --- | --- | --- | --- | --- | --- | --- | --- | --- | --- | --- | --- |
| *Advice condition* | | | *Concurrent advice* | | | *On-demand advice* | | | |  | | |  |  |  |  |  |  |
| Mean | | | 89.27 | | | 88.61 | | | |  | | |  |  |  |  |  |  |
| SD | | | 7.02 | | | 9.09 | | | |  | | |  |  |  |  |  |  |
|  | | |  | | |  | | | |  | | |  |  |  |  |  |  |
| *Advice condition* | | | *Concurrent advice* | | | | | *On-demand advice* | | | |  | |  |  |  |  |  |
| *AI prediction* | | | *accurate* | | *inaccurate* | | | *accurate* | | | *inaccurate* |  | |  |  |  |  |  |
| Mean | | | 91.17 | | 65.83 | | 90.10 | | | | 70.21 |  | |  |  |  |  |  |
| SD | | | 6.88 | | 23.56 | | 8.68 | | | | 23.52 |  | |  |  |  |  |  |
|  | | |  |  | | |  | |  | | |  | |  | |  |  |  |
|  | | |  |  | | |  | |  | | |  | |  | |  |  |  |
| **Experiment 1b** | | |  |  | | |  | |  | | |  | |  | |  |  |  |
| *Advice condition* | | | *Concurrent similarity ratings* | | | | *On-demand similarity ratings* | | | | |  | | |  |  |  |  |
| Mean | | | 88.43 | | | | 88.51 | | | | |  | | |  |  |  |  |
| SD | | | 8.51 | | | | 7.73 | | | | |  | | |  |  |  |  |
|  | | |  |  | | |  | |  | | |  | |  | |  |  | |
| *Advice condition* | | | *Concurrent similarity ratings* | | | | *On-demand similarity ratings* | | | | |  | | |  |  |  |  |
| *AI prediction* | | | *accurate* | *inaccurate* | | | *accurate* | | *inaccurate* | | |  | |  |  |  |  |  |
| Mean | | | 90.44 | 63.64 | | | 90.45 | | 64.50 | | |  | | |  |  |  |  |
| SD | | | 8.07 | 27.40 | | | 7.88 | | 25.70 | | |  | | |  |  |  |  |
|  |  |  |  |  |  |  |  |  |  |  |  |  |  |  |  |  |  |  |
|  | | |  |  | | |  | |  | | |  | |  | |  |  |  |
| **Experiment 2** | | |  |  | | |  | |  | | |  | |  | |  |  |  |
| *Advice condition* | | | *No advice* | | | | *Concurrent advice* | | | | |  | | *Conditional advice* | |  |  |  |
| Mean | | | 87.91 | | | | 92.30 | | | | |  | | 93.17 | |  |  |  |
| SD | | | 11.82 | | | | 7.10 | | | | |  | | 6.03 | |  |  |  |
|  | | |  |  | | |  | |  | | |  | |  | |  |  |  |
| *Advice condition* | | | *Concurrent advice* | | | | *Conditional advice* | | | | |  | |  | |  |  |  |
| *AI prediction* | | | *accurate* | *inaccurate* | | | *accurate* | | *inaccurate* | | | | |  | |  |  |  |
| Mean | | | 93.97 | 71.71 | | | 95.66 | | 62.40 | | |  | |  | |  |  |  |
| SD | | | 6.17 | 32.14 | | | 5.70 | | 34.22 | | |  | |  | |  |  |  |

#

# Supplementary Results: Experiment 1a

# *eTable 2*: Logistic mixed effects regression models for participants’ agreement with AI advice

|  | **AI agreement** | | | | | **AI agreement** | | | | |
| --- | --- | --- | --- | --- | --- | --- | --- | --- | --- | --- |
| *Predictors* | *Odds Ratios* | *SE* | *95% CI* | *Statistic* | *p* | *Odds Ratios* | *SE* | *95% CI* | *Statistic* | *p* |
| Intercept | 16.49 | 2.74 | 11.91 – 22.84 | 16.87 | **<0.001** | 20.04 | 2.85 | 15.17 – 26.49 | 21.07 | **<0.001** |
| Advice condition [on-demand] | 1.52 | 0.16 | 1.24 – 1.87 | 3.97 | **<0.001** | 1.48 | 0.17 | 1.18 – 1.86 | 3.38 | **0.001** |
| Prediction accuracy [incorrect] |  |  |  |  |  | 0.02 | 0.01 | 0.01 – 0.05 | -9.62 | **<0.001** |
| Advice condition [on-demand] x Prediction accuracy [incorrect] |  |  |  |  |  | 1.09 | 0.30 | 0.64 – 1.86 | 0.32 | 0.747 |
| **Random Effects** | | | | | | | | | | |
| σ^2^ | 3.29 | | | | | 3.29 | | | | |
| τ_00_ | 2.84 _TrialID_ | | | | | 1.49 _TrialID_ | | | | |
|  | 0.44 _participant_ | | | | | 0.45 _participant_ | | | | |
| ICC | 0.50 | | | | | 0.37 | | | | |
| N | 160 _TrialID_ | | | | | 160 _TrialID_ | | | | |
|  | 80 _participant_ | | | | | 80 _participant_ | | | | |
| Observations | 8088 | | | | | 8088 | | | | |
| Marginal R^2^ / Conditional R^2^ | 0.004 / 0.502 | | | | | 0.163 / 0.473 | | | | |

# *Note:* OR > 1 is associated with higher odds for correct decision; OR < 1 is associated with lower odds for correct decision.

# Supplementary Results: Experiment 1b

### We examined whether performance differed by trial type (i.e. match trials vs. mismatch trials). A mixed-effects logistic regression with trial type as a fixed factor showed that participants performed worse overall on mismatch compared to match trials (eTable 3). We further examined the interaction of trial type and advice condition, which again showed a main effect of trial type, with participants performing worse on mismatch trials. A main effect of advice condition or a significant interaction were not observed. This is a commonly reported phenomenon in the face matching literature, even without AI involvement (Burton et al., 2010; Kokje et al., 2018; Tummon et al., 2019), as well as with AI involvement (Fysh & Bindemann, 2018). This is because the cognitive processes for classifying matches and mismatches are distinct and dissociated from each other and is stimulus-dependent (Fysh & Bindemann, 2023).

Further, we explored whether the higher rate of demanding similarity ratings for the incorrect advice cases (19.91% vs. 12%) potentially reflected item difficulty, which influenced both AI and human observers. As we did not have a direct measure of item difficulty, we used two measures as proxies. First, we used the mean performance of participants on each face pair as a proxy, and conducted a regression analysis with the performance on the face pair as the predictor and the rate of demanding similarity ratings for the face pair as the outcome. We found that performance significantly predicted the rate of demanding similarity ratings (*R^2^* = .18, *F(1, 158)* = 33.52, *p* < .001), such that the better the performance on a face pair, the less likely participants were to demand the similarity rating for it (eFigure 2).

**
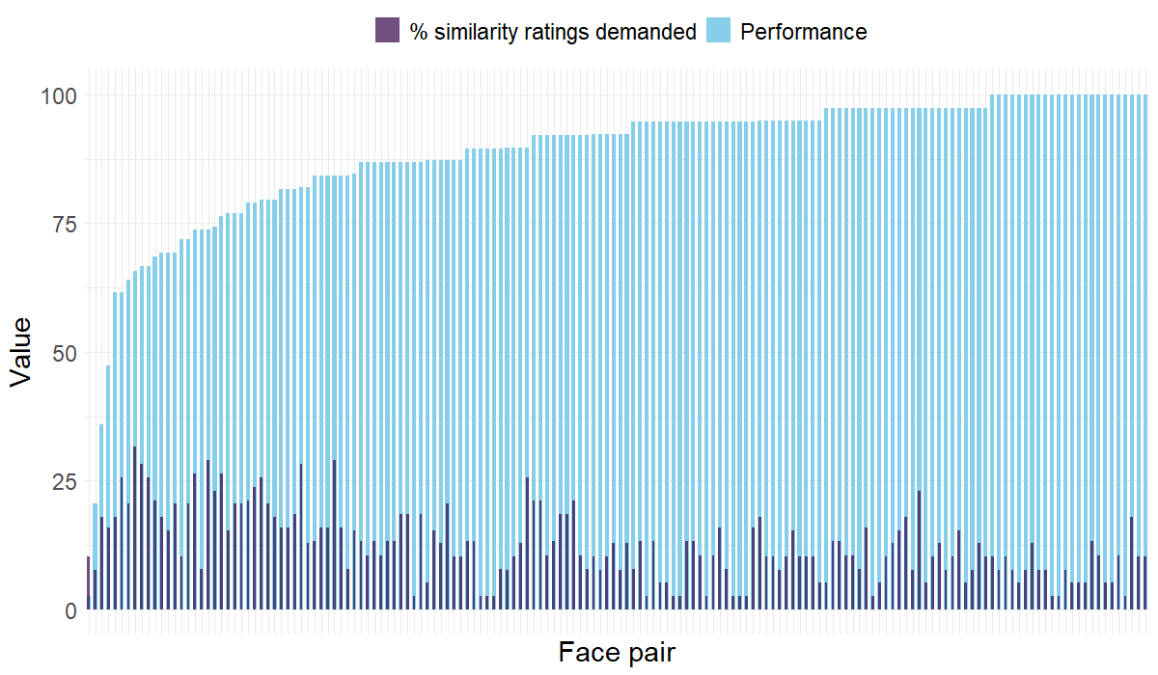
**

*eFigure 2:* Distribution of mean performance and mean rate of demanding similarity ratings for each face pair.

Second, we used the similarity value as a proxy for difficulty, and found that it also significantly predicted the rate of demanding similarity ratings (*R^2^* = .07, *F(1, 158)* = 11.60, *p* < .001). For the algorithm, we examined the distribution of the similarity values for correct and incorrect AI predictions (see eFigure 3). The values for the correct advice cases showed a bimodal distribution with values farther away from the match/mismatch classification threshold, whereas the values for incorrect advice cases clustered mostly around the threshold, indicating that the incorrectly classified cases were indeed more ambiguous or difficult for the algorithm.


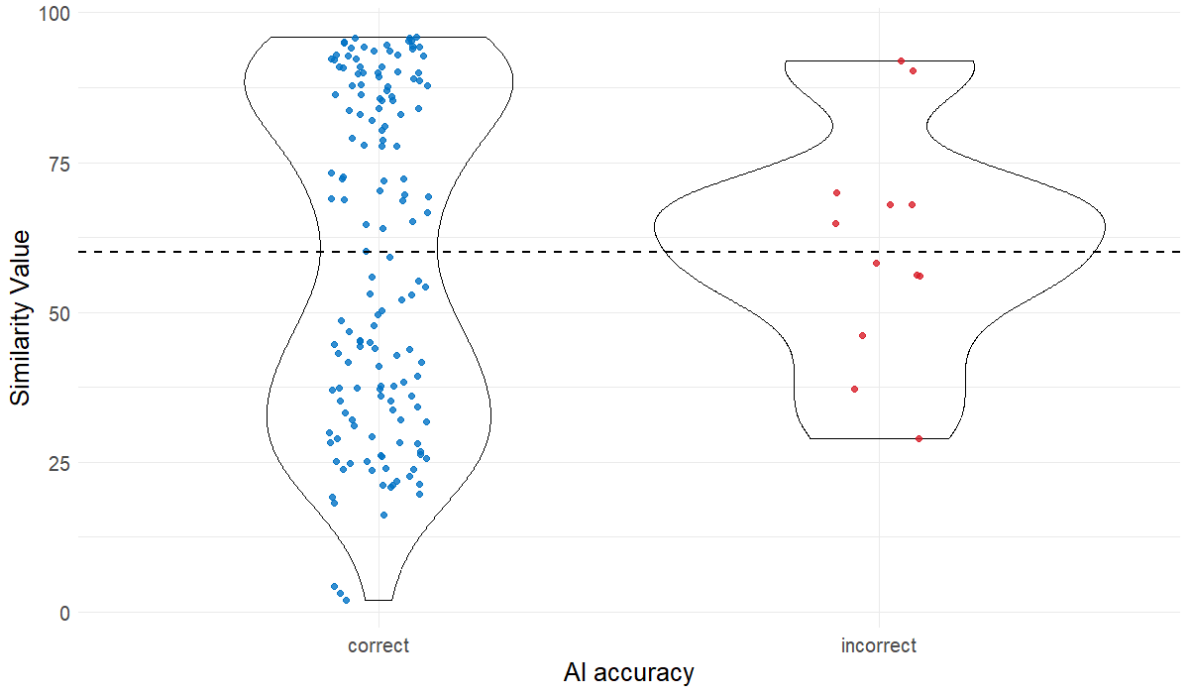


*eFigure 3:* Distribution of the similarity values generated by the algorithm for correct and incorrect advice cases. Dotted line represents the match/mismatch classification threshold (60).

We also conducted analyses to examine individual differences in performance (and relative to the AI performance) and tendency to demand similarity ratings. First, we conducted a linear regression to check whether individual performance in the on-demand similarity ratings condition predicted the rate of demanding similarity ratings, but found it was not significant (*R^2^* = .008, *F(1, 75)* = 0.64, *p* = .426). To examine performance relative to AI performance, we clustered participants according to their performance in the on-demand similarity ratings condition into three groups: *better than AI (> 92.5%)*, *equal to AI (92.5%),* or *worse than AI (< 92.5%).* A linear regression showed that participants’ performance relative to AI did not significantly predict their tendency to demand similarity ratings (*R^2^* = .03, *F(2, 74)* = 0.98, *p* = .379).

In conclusion, the pattern for demanding similarity ratings does appear to reflect item difficulty for participants and for AI. However, individual differences in performance did not influence the tendency to demand similarity ratings.

# *eTable 3*: Logistic mixed effects regression models for participants’ performance

|  | **Performance** | | | | | **Performance** | | | | |
| --- | --- | --- | --- | --- | --- | --- | --- | --- | --- | --- |
| *Predictors* | *Odds Ratios* | *SE* | *95% CI* | *Statistic* | *p* | *Odds Ratios* | *SE* | *95% CI* | *Statistic* | *p* |
| Intercept | 26.77 | 5.30 | 18.17 – 39.46 | 16.61 | **<0.001** | 27.09 | 5.53 | 18.15 – 40.42 | 16.15 | **<0.001** |
| Trial type [mismatch] | 0.44 | 0.10 | 0.28 – 0.67 | -3.72 | **<0.001** | 0.43 | 0.10 | 0.27 – 0.67 | -3.67 | **<0.001** |
| Advice condition [on-demand] |  |  |  |  |  | 0.98 | 0.10 | 0.80 – 1.19 | -0.23 | 0.815 |
| Trial type [mismatch] x Advice condition [on-demand] |  |  |  |  |  | 1.05 | 0.14 | 0.81 – 1.36 | 0.36 | 0.720 |
| **Random Effects** | | | | | | | | | | |
| σ^2^ | 3.29 | | | | | 3.29 | | | | |
| τ_00_ | 1.74 _TrialID_ | | | | | 1.74 _TrialID_ | | | | |
|  | 0.96 _participant_ | | | | | 0.96 _participant_ | | | | |
| ICC | 0.45 | | | | | 0.45 | | | | |
| N | 160 _TrialID_ | | | | | 160 _TrialID_ | | | | |
|  | 77 _participant_ | | | | | 77 _participant_ | | | | |
| Observations | 12320 | | | | | 12320 | | | | |
| Marginal R^2^ / Conditional R^2^ | 0.028 / 0.466 | | | | | 0.028 / 0.466 | | | | |

*Note:* OR > 1 is associated with higher odds for correct decision; OR < 1 is associated with lower odds for correct decision.

# *eTable 4*: Logistic mixed effects regression models for participants’ performance

|  | **Performance** | | | | | **Performance** | | | | |
| --- | --- | --- | --- | --- | --- | --- | --- | --- | --- | --- |
| *Predictors* | *Odds Ratios* | *SE* | *95% CI* | *Statistic* | *p* | *Odds Ratios* | *SE* | *95% CI* | *Statistic* | *p* |
| Intercept | 17.65 | 2.95 | 12.72 – 24.51 | 17.15 | **<0.001** | 20.93 | 3.42 | 15.20 – 28.84 | 18.61 | **<0.001** |
| Advice condition [on-demand] | 1.00 | 0.06 | 0.89 – 1.14 | 0.06 | 0.948 | 0.99 | 0.07 | 0.86 – 1.13 | -0.18 | 0.855 |
| Advice accuracy [incorrect] |  |  |  |  |  | 0.09 | 0.04 | 0.04 – 0.20 | -6.05 | **<0.001** |
| Advice condition [on-demand] x Advice accuracy [incorrect] |  |  |  |  |  | 1.14 | 0.22 | 0.78 – 1.66 | 0.69 | 0.491 |
| **Random Effects** | | | | | | | | | | |
| σ^2^ | 3.29 | | | | | 3.29 | | | | |
| τ_00_ | 1.92 _TrialID_ | | | | | 1.50 _TrialID_ | | | | |
|  | 0.96 _participant_ | | | | | 0.97 _participant_ | | | | |
| ICC | 0.47 | | | | | 0.43 | | | | |
| N | 160 _TrialID_ | | | | | 160 _TrialID_ | | | | |
|  | 77 _participant_ | | | | | 77 _participant_ | | | | |
| Observations | 12320 | | | | | 12320 | | | | |
| Marginal R^2^ / Conditional R^2^ | 0.000 / 0.467 | | | | | 0.061 / 0.463 | | | | |

*Note:* OR > 1 is associated with higher odds for correct decision; OR < 1 is associated with lower odds for correct decision.

# *eTable 5*: Logistic mixed effects regression models for participants’ agreement with AI advice

|  | **AI agreement** | | | | | **AI agreement** | | | | |
| --- | --- | --- | --- | --- | --- | --- | --- | --- | --- | --- |
| *Predictors* | *Odds Ratios* | *SE* | *95% CI* | *Statistic* | *p* | *Odds Ratios* | *SE* | *95% CI* | *Statistic* | *p* |
| Intercept | 15.16 | 2.51 | 10.95 – 20.97 | 16.40 | **<0.001** | 18.89 | 2.79 | 14.14 – 25.24 | 19.87 | **<0.001** |
| on-demand [No] | 1.06 | 0.07 | 0.93 – 1.20 | 0.82 | 0.411 | 1.06 | 0.08 | 0.92 – 1.22 | 0.80 | 0.422 |
| on-demand [Yes] | 0.66 | 0.09 | 0.51 – 0.86 | -3.15 | **0.002** | 0.68 | 0.10 | 0.51 – 0.89 | -2.76 | **0.006** |
| Advice accuracy [incorrect] |  |  |  |  |  | 0.03 | 0.01 | 0.01 – 0.06 | -9.33 | **<0.001** |
| on-demand [No] x Advice accuracy [incorrect] |  |  |  |  |  | 0.97 | 0.20 | 0.66 – 1.45 | -0.13 | 0.900 |
| on-demand [Yes] x Advice accuracy [incorrect] |  |  |  |  |  | 0.85 | 0.29 | 0.44 – 1.64 | -0.50 | 0.618 |
| **Random Effects** | | | | | | | | | | |
| σ^2^ | 3.29 | | | | | 3.29 | | | | |
| τ_00_ | 2.50 _TrialID_ | | | | | 1.41 _TrialID_ | | | | |
|  | 0.66 _participant_ | | | | | 0.67 _participant_ | | | | |
| ICC | 0.49 | | | | | 0.39 | | | | |
| N | 160 _TrialID_ | | | | | 160 _TrialID_ | | | | |
|  | 77 _participant_ | | | | | 77 _participant_ | | | | |
| Observations | 12320 | | | | | 12320 | | | | |
| Marginal R^2^ / Conditional R^2^ | 0.002 / 0.491 | | | | | 0.147 / 0.477 | | | | |

# Supplementary Results: Experiment 1 (cross-experiment)


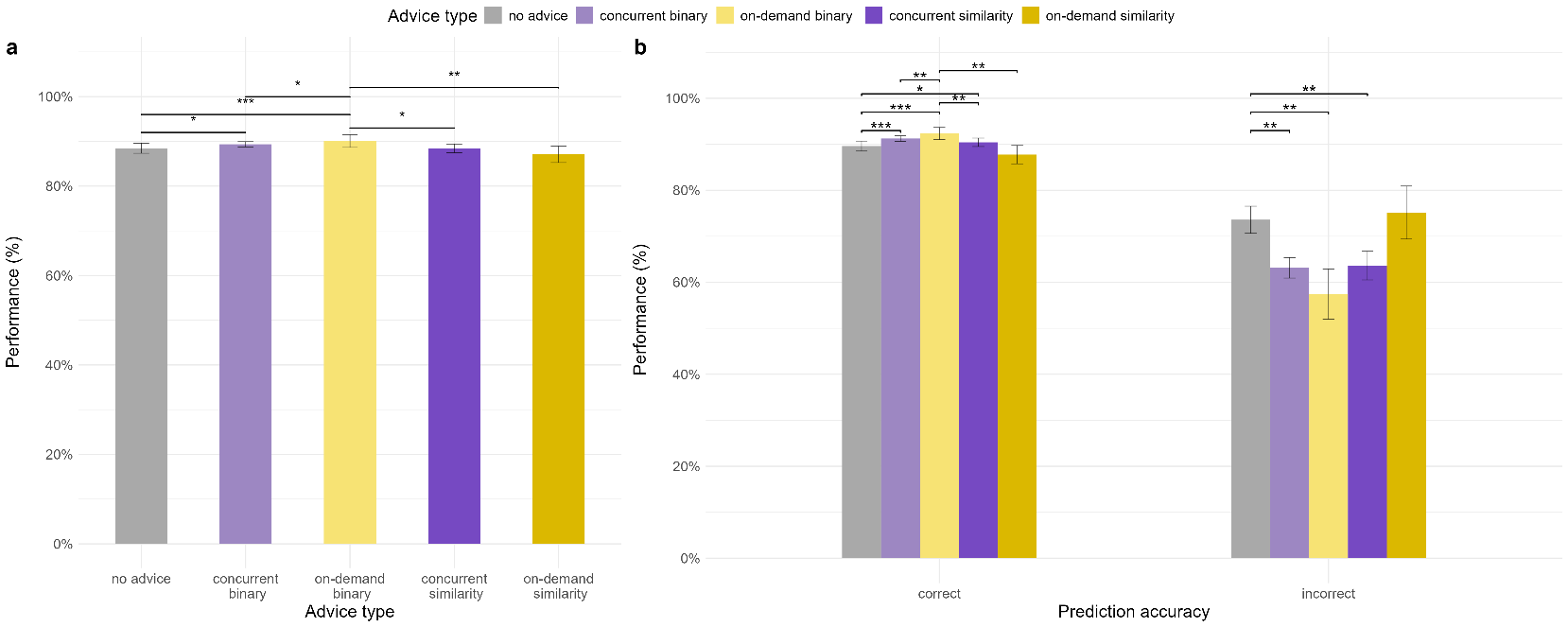


*eFigure 4:* Performance in the binary advice and similarity rating conditions across Experiments 1a and 1b (a) per advice type, and (b) per advice type and accuracy of prediction.

*Note:* Default binary advice includes the ‘concurrent advice condition’ in Experiment 1a and the ‘on-demand similarity (No) condition’ in Experiment 1b; only significant comparisons are highlighted.

# Supplementary Results: Experiment 2


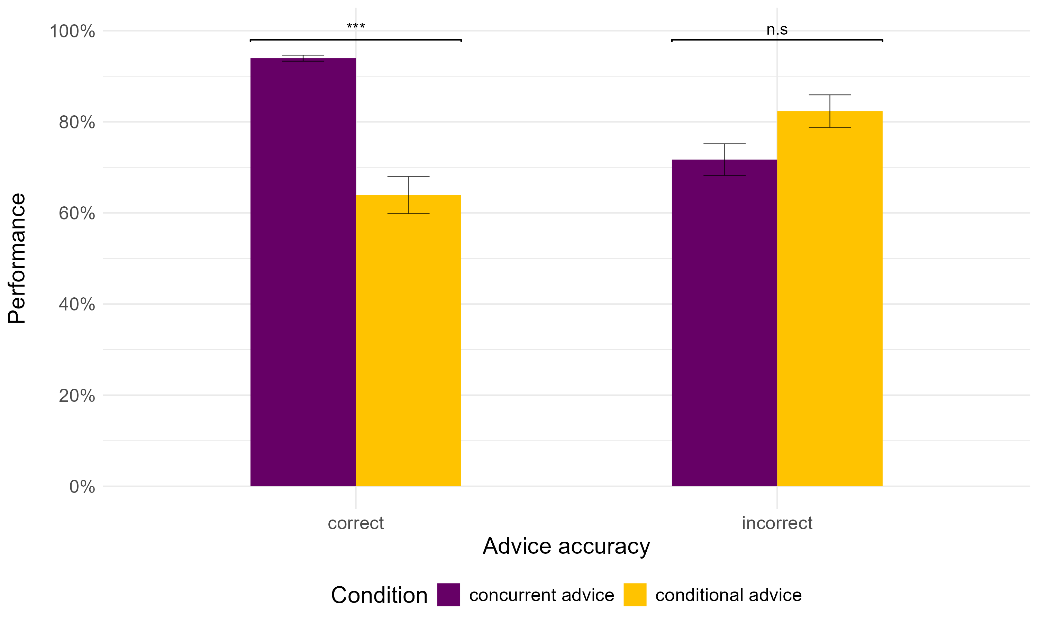


*eFigure 5:* Performance in the two advice conditions on trials where advice was presented.

|  | **AI agreement** | | | | | **AI agreement** | | | | |
| --- | --- | --- | --- | --- | --- | --- | --- | --- | --- | --- |
| *Predictors* | *Odds Ratios* | *SE* | *95% CI* | *Statistic* | *p* | *Odds Ratios* | *SE* | *95% CI* | *Statistic* | *p* |
| Intercept | 19.18 | 3.50 | 13.42 – 27.42 | 16.20 | **<0.001** | 23.50 | 3.36 | 17.77 – 31.10 | 22.10 | **<0.001** |
| Advice condition [Conditional] | 0.20 | 0.03 | 0.15 – 0.26 | -11.52 | **<0.001** | 0.13 | 0.02 | 0.10 – 0.18 | -13.16 | **<0.001** |
| Prediction accuracy [incorrect] |  |  |  |  |  | 0.01 | 0.01 | 0.01 – 0.03 | -11.28 | **<0.001** |
| Advice condition [Conditional] x Prediction accuracy [incorrect] |  |  |  |  |  | 5.56 | 1.73 | 3.02 – 10.24 | 5.51 | **<0.001** |
| **Random Effects** | | | | | | | | | | |
| σ^2^ | 3.29 | | | | | 3.29 | | | | |
| τ_00_ | 2.41 _TrialID_ | | | | | 0.85 _TrialID_ | | | | |
|  | 0.24 _participant_ | | | | | 0.22 _participant_ | | | | |
| ICC | 0.45 | | | | | 0.25 | | | | |
| N | 120 _TrialID_ | | | | | 120 _TrialID_ | | | | |
|  | 86 _participant_ | | | | | 86 _participant_ | | | | |
| Observations | 3970 | | | | | 3970 | | | | |
| Marginal R^2^ / Conditional R^2^ | 0.048 / 0.473 | | | | | 0.323 / 0.490 | | | | |

# *eTable 6*: Logistic mixed effects regression models for participants’ agreement with AI advice

*Note:* OR > 1 is associated with higher odds for correct decision; OR < 1 is associated with lower odds for correct decision.

# *eTable 7*: Ordinal mixed effects regression models for participants’ confidence in their decision

|  | **confidence** | | | | | | **confidence** | | | | |
| --- | --- | --- | --- | --- | --- | --- | --- | --- | --- | --- | --- |
| *Predictors* | *Odds Ratios* | *SE* | *95% CI* | | *Statistic* | *p* | *Odds Ratios* | *SE* | *95% CI* | *Statistic* | *p* |
| 1\|2 | 0.00 | 0.00 | 0.00 – 0.01 | | -13.38 | **<0.001** | 0.01 | 0.00 | 0.00 – 0.02 | -11.64 | **<0.001** |
| 2\|3 | 0.02 | 0.01 | 0.01 – 0.03 | | -13.22 | **<0.001** | 0.03 | 0.01 | 0.02 – 0.06 | -10.80 | **<0.001** |
| 3\|4 | 0.05 | 0.01 | 0.03 – 0.09 | | -10.95 | **<0.001** | 0.10 | 0.03 | 0.06 – 0.18 | -8.17 | **<0.001** |
| 4\|5 | 0.23 | 0.06 | 0.14 – 0.37 | | -6.10 | **<0.001** | 0.44 | 0.11 | 0.27 – 0.73 | -3.22 | **0.001** |
| 5\|6 | 1.77 | 0.42 | 1.12 – 2.81 | | 2.45 | **0.014** | 3.42 | 0.86 | 2.09 – 5.59 | 4.90 | **<0.001** |
| 6\|7 | 11.10 | 3.01 | 6.52 – 18.90 | | 8.86 | **<0.001** | 21.86 | 6.38 | 12.33 – 38.74 | 10.56 | **<0.001** |
| Switch [yes] | 0.38 | 0.08 | 0.25 – 0.57 | | -4.60 | **<0.001** | 0.66 | 0.16 | 0.42 – 1.05 | -1.74 | 0.081 |
| Advice accuracy [incorrect] |  |  |  | |  |  | 5.07 | 1.47 | 2.87 – 8.94 | 5.61 | **<0.001** |
| Switch [yes] x Advice accuracy [incorrect] |  |  |  | |  |  | 0.20 | 0.09 | 0.08 – 0.50 | -3.49 | **<0.001** |
| **Random Effects** | | | |  |  |  |  |  |  |  |  |
| σ^2^ | 3.29 | 3.29 | |  |  |  |  |  |  |  |  |
| τ_00_ | 0.37 _TrialID_ | 0.12 _TrialID_ | |  |  |  |  |  |  |  |  |
|  | 1.72 _participant_ | 1.56 _participant_ | |  |  |  |  |  |  |  |  |
| ICC | 0.39 | 0.34 | |  |  |  |  |  |  |  |  |
| N | 99 _TrialID_ | 99 _TrialID_ | |  |  |  |  |  |  |  |  |
|  | 86 _participant_ | 86 _participant_ | |  |  |  |  |  |  |  |  |
| Observations | 530 | 530 | |  |  |  |  |  |  |  |  |
| Marginal R^2^ / Conditional R^2^ | 0.041 / 0.414 | 0.135 / 0.427 | |  |  |  |  |  |  |  |  |

*Note:* OR > 1 is associated with higher odds for correct decision; OR < 1 is associated with lower odds for correct decision.

# References

Burton, A. M., White, D., & McNeill, A. (2010). The Glasgow Face Matching Test. *Behavior Research Methods*, *42*(1), 286–291. https://doi.org/10.3758/BRM.42.1.286

Fysh, M. C., & Bindemann, M. (2018). Human–Computer Interaction in Face Matching. *Cognitive Science*, *42*(5), 1714–1732. https://doi.org/10.1111/cogs.12633

Fysh, M. C., & Bindemann, M. (2023). Understanding face matching. *Quarterly Journal of Experimental Psychology*, *76*(4), 862–880. https://doi.org/10.1177/17470218221104476

Kokje, E., Bindemann, M., & Megreya, A. M. (2018). Cross-race correlations in the abilities to match unfamiliar faces. *Acta Psychologica*, *185*, 13–21. https://doi.org/10.1016/j.actpsy.2018.01.006

Schroff, F., Kalenichenko, D., & Philbin, J. (2015). FaceNet: A unified embedding for face recognition and clustering. *2015 IEEE Conference on Computer Vision and Pattern Recognition (CVPR)*, 815–823. https://doi.org/10.1109/CVPR.2015.7298682

Tummon, H. M., Allen, J., & Bindemann, M. (2019). Facial Identification at a Virtual Reality Airport. *I-Perception*, *10*(4), 2041669519863077. https://doi.org/10.1177/2041669519863077
